# Biomarkers in Cancer Detection, Diagnosis, and Prognosis

**DOI:** 10.3390/s24010037

**Published:** 2023-12-20

**Authors:** Sreyashi Das, Mohan Kumar Dey, Ram Devireddy, Manas Ranjan Gartia

**Affiliations:** Department of Mechanical and Industrial Engineering, Louisiana State University, Baton Rouge, LA 70803, USA; sreyashidas143@gmail.com (S.D.); mdey1@lsu.edu (M.K.D.); rdevir1@lsu.edu (R.D.)

**Keywords:** biomarkers, DNA, RNA, lipids, PET, MRI, CT needle biopsy, RNAseq, proteomics

## Abstract

Biomarkers are vital in healthcare as they provide valuable insights into disease diagnosis, prognosis, treatment response, and personalized medicine. They serve as objective indicators, enabling early detection and intervention, leading to improved patient outcomes and reduced costs. Biomarkers also guide treatment decisions by predicting disease outcomes and facilitating individualized treatment plans. They play a role in monitoring disease progression, adjusting treatments, and detecting early signs of recurrence. Furthermore, biomarkers enhance drug development and clinical trials by identifying suitable patients and accelerating the approval process. In this review paper, we described a variety of biomarkers applicable for cancer detection and diagnosis, such as imaging-based diagnosis (CT, SPECT, MRI, and PET), blood-based biomarkers (proteins, genes, mRNA, and peptides), cell imaging-based diagnosis (needle biopsy and CTC), tissue imaging-based diagnosis (IHC), and genetic-based biomarkers (RNAseq, scRNAseq, and spatial transcriptomics).

## 1. Introduction

A biomarker is a biological phenomenon that can be difficult to find, yet indicates a clinically significant outcome or interim consequence. Biomarker applications include identifying, characterizing, and monitoring diseases. Additionally, biomarkers can act as prognostic indicators, inform individualized treatment plans, and anticipate and manage negative medication reactions. Understanding the fundamental link between a biomarker and its clinical result is crucial for adequately appreciating its significance [1].

The National Cancer Institute defines biomarkers as biological molecules in blood, bodily fluids, or tissues that reveal whether a process, condition, or disease—such as cancer—is normal or aberrant [2]. They are essential for identifying those with and without the disease, and changes in them can be attributed to genetic mutations, transcriptional alterations, and post-translational modifications [3]. Proteins, nucleic acids, antibodies, and peptides are only a few of the many molecules that make up a biomarker. Gene expression patterns, proteomic profiles, and metabolomic signatures are only a few examples of the combinations of modifications they can include. Biomarkers may need a tissue sample taken through biopsy or imaging, or they may be identified non-invasively through blood, urine, saliva, sweat, cerebrospinal fluid (CSF), or other bodily fluids [4]. For each of these uses, this review offers concrete examples of biomarkers. However, contrast between biomarkers and targets for therapy must be understood, as they are not identical [5].

Cancer is an intricate condition marked by genetic and epigenetic changes that throw off the balance between cellular development and cell death. It is a major global health issue that kills many people every year worldwide [6]. Significant molecular and tissue alterations are required for cancer growth. Invaluable clinical data in the form of biomarkers can be generated by analyzing biomolecules such as nucleic acids, carbohydrates, proteins, lipids, and metabolites linked to cancer development [7]. Early detection plays a crucial role in minimizing the morbidity and mortality associated with cancer. Therefore, there is an urgent need for genuine and reliable cancer indicators. PSA, CEA, and CA-125/MUC16 are frequently used cancer indicators, while exosomes, microRNA, and circulating tumor cells are emerging as a new source of biomarkers [8].

There are several factors to consider and difficulties to overcome while developing and using biomarkers in healthcare settings. Analytical validity, clinical validity, and clinical utility are among the stages and elements that create a possible biomarker [9,10]. Analytic validity concerns pre-analytical and analytical aspects of the biomarker assay, such as sample handling and assay accuracy. Clinical validity tests how well the biomarker can identify distinct populations within the target population, and necessitates independent validation. Given the effectiveness of the biomarker and the balance between potential advantages and risks, clinical utility suggests that there is strong evidence to justify its usage in patient treatment [10,11,12]. This review paper discusses the challenges associated with cancer detection, the conventional mode of cancer detection, various types of biomarkers, and their roles in cancer detection.

## 2. Challenges Associated with Detecting Early-Stage Tumors

Successful cancer treatment depends on early detection [13]. Yet, physiological and mass transport barriers restrict the amount of biological indicators that can be released from early lesions [14,15].

**Figure 1:**
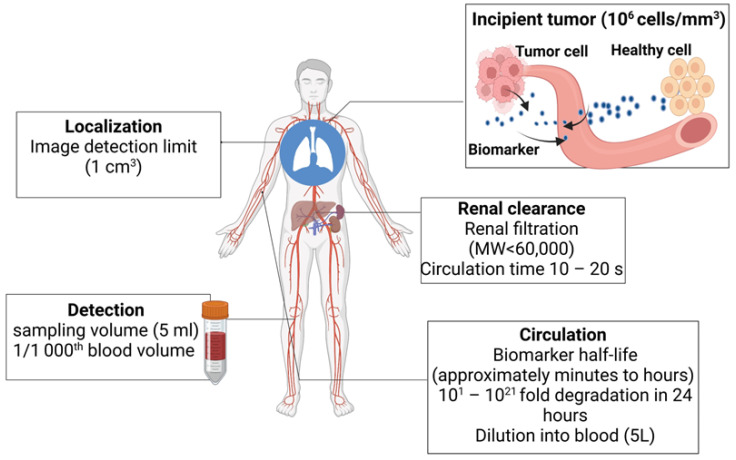
Difficulties related to the identification of tumors in their early stages. Due to their tiny size and the difficulties in transferring biomarkers from the tumor microenvironment to the bloodstream, early-stage cancers are challenging to detect. This is brought on by difficulties with biomarker transfer, dilution, and the kidneys’ quick degradation and filtration processes. Only a few tumor-associated biomarkers can be found in a typical blood sample of 5–10 mL, which is a small part of the overall blood volume.

Finding intrinsic biomarkers through blood and biofluid examination is the primary objective of ongoing research. To improve specificity, bioengineered sensors and synthetic markers are being developed. Imaging systems also aid in detecting and localizing tumors [16,17,18]. The typical spatial resolution of a positron emission tomography (PET) scanner is about 1 cm^3^, and hence, very small tumors (diameter < 5 mm) will be missed by the PET imagers. The typical blood draw is 5–10 mL, which is three orders of magnitude (1/1000th) smaller than the body’s total blood volume (~5 L). This means that the biomarkers shed by the tumor will be diluted > 1000 times when it is detected (Figure 1). Further, there are challenges in detecting genomic materials. For example, circulating tumor DNA (ctDNA) has a half-life of ~1.5 h. So, in a 24 h time period, it will undergo 16 half-lives. This means that by the time it is detected, only 0.0015% of the original materials will remain [17,18,19,20]. A potential ten-year window for early cancer detection is suggested by multicompartment models and studies on the genomic timeline. However, current screening techniques can find cancers that have been present for ten years or longer and are indolent. Cancers that spread quickly and aggressively and have poor clinical outcomes include triple-negative breast cancer and high-grade serous ovarian carcinoma. These problems are intended to be solved by activity-based or genetically encoded mechanisms for early detection in synthetic biomarker research.

## 3. Biomarkers in Cancer Detection, Diagnosis, and Prognosis

Genetic alterations that encourage cell survival and proliferation are what produces the uncontrolled cell growth that defines cancer [19]. By interfering with cell death mechanisms and cell proliferation, alterations in the genes responsible for tumor suppression, DNA repair, and proto-oncogenes aid in the growth of cancer. Cancer development is also influenced by epigenetic alterations such as DNA methylation and altered histone patterns. In this section, we discuss different categories of biomarkers and their mode of detection.

### 3.1. Biofluid Biomarkers

Biofluids provide a way to quickly evaluate and track diseases [20]. Urine, saliva, blood, and sweat are examples of biofluids that contain important data regarding the disease under investigation. These biofluid specimens can be easily collected non-invasively and are ideal for clinical studies [21]. Each biofluid has unique advantages and challenges [22]. Saliva is readily available and includes electrolytes like sodium, potassium, calcium, magnesium, bicarbonate, and phosphates, whereas urine contains urea, chloride, sodium, and potassium salts. Sweat mainly contains sodium, chloride, minerals, lactic acid, and urea [23].

Cancer detection and tracking uses various biofluids, such as urine, saliva, blood, and cerebrospinal fluid (CSF) [24]. Studies have identified KRAS, MBD3L2, ACRV1, and DPM1 as biomarkers in salivary mRNA to detect pancreatic cancer with high specificity [25,26,27,28,29]. Salivary proteins with high specificity and sensitivity to identify lung cancer include calprotectin, AZGP1, and HP. Salivary DNA can also detect mutations in the genes PI3K, CDKN2A, FBXW7, HRAS, and KRAS in mouth and throat tumors [30,31,32,33,34].

**Figure 2:**
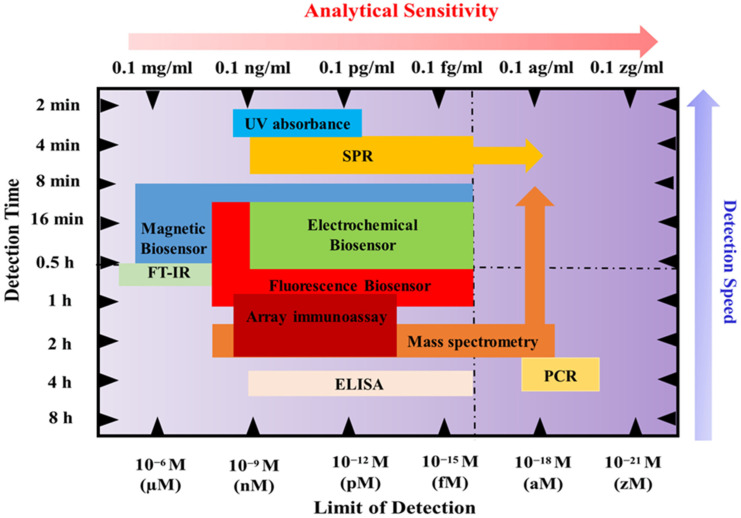
The diagram shows the analytical sensitivity and detection times of various biosensing techniques.

There are various techniques to detect genomics (qPCR, RNA, and DNA sequencing), proteomics (mass spectrometry, ELISA, and Western blotting), and lipidomics (mass spectrometry) to find cancer biomarkers in biofluids. For protein extraction and separation, several methods are used, including surface-enhanced laser desorption/ionization (SELDI), 2-dimensional gel electrophoresis (2-DE), difference gel electrophoresis (2D-DIGE), and Liquid Chip. Mass spectrometry and bioinformatics are used to identify proteins, with Western blot and ELISA used to confirm the results. Sample variability, inter-laboratory analytical variability, and sample type selection are difficulties in biomarker discovery. Many studies have looked into finding cancer-associated hypermethylated DNA fragments in cancer patients’ circulating free DNA (cfDNA), especially in cases of gastric cancer (GC). A number of hypermethylated genes, including RPRM, XAF1, and a KCNA4 and CYP26B1 combination, have demonstrated high diagnostic value for GC detection. Before these assays can be used in clinical settings, a few technical issues must be resolved. The majority of studies employ sodium bisulfite treatment followed by methylation-specific PCR (MSP) or DNA sequencing, but these methods could produce false-positive results because unmethylated cytosine residues are not completely converted. The sensitivities of various biofluid detection techniques are shown in Figure 2.

### 3.2. Imaging Biomarkers

Tumor, node, metastasis (TNM) staging, objective response, and left ventricular ejection fraction are just a few of the imaging biomarkers (IBs) that are critical for clinical oncology [35]. Cancer research frequently uses imaging techniques like computed tomography (CT), magnetic resonance imaging (MRI), positron emission tomography (PET), and ultrasonography. New IBs need to be validated and qualified in order to fill in the translational gaps [36]. A total of 14 important recommendations have been made by Cancer Research UK (CRUK) and the European Organization for Research and Treatment of Cancer (EORTC) to hasten the clinical translation of IBs [37,38,39]. These suggestions for achieving IB qualification emphasize parallel validation procedures, cost-effectiveness analysis, standardization, accreditation systems, precision evaluation, alternative validation frameworks, and multicenter studies [40,41,42,43,44,45].

IBs are derived from medical images. They offer non-invasive, cost-effective screening, tumor detection, patient progress, and therapy response monitoring tools [46]. Staging systems document the existence, dimensions, and quantity of abnormalities in tumor, lymph node, and additional metastatic locations to establish a structured categorical indicator of the patient’s disease severity. IBs have the ability to map tumor heterogeneity, monitor changes in tumors over time, and assess a person’s multiple lesions [47,48,49].

**Figure 3:**
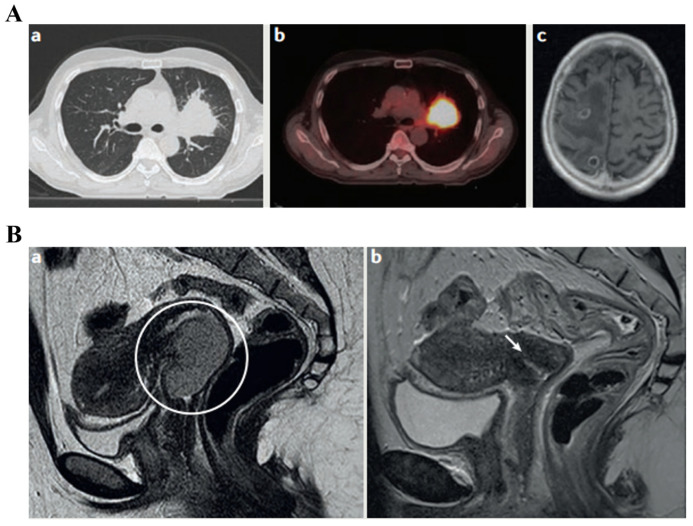
(**A**) The TNM staging of a patient diagnosed with stage IV non-small-cell lung cancer (T2 N0 M1) revealed the following: (**a**) a T2 tumor in the lung detected through CT imaging, (**b**) no signs of involvement in local lymph nodes based on PET-CT scans, and (**c**) the presence of brain metastases as observed in MRI scans. (**B**) (**a**) An individual with cervical cancer (T3b N0 M0) initially came with a sizable main tumor (shown inside the circle). (**b**) Nevertheless, following chemoradiation therapy, the patient showed a full recovery, and the cervix was returned to normal (the location of the remnant tumor is shown with the arrow) [51].

The evaluation of lesions at tumor, nodal, and metastatic sites using staging systems is crucial for the diagnosis and prognosis of cancer. The American Joint Committee on Cancer (AJCC) offers recommendations for precise and consistent reporting in radiology. TNM staging is frequently used and has prognostic value for a variety of cancer types. It is based on imaging modalities like CT, MRI, SPECT, and PET. TNM staging can occasionally be used to forecast treatment outcomes. For instance, the clinical TNM stage in prostate cancer serves as a predictive biomarker for the efficacy of bicalutamide monotherapy by differentiating between localized and locally advanced diseases [50]. IBs underwent successful translation and are now applied in clinical settings. Solid tumors are evaluated using response criteria like RECIST 1.0 and 1.1, WHO, and RECIST 1.0. A popular biomarker called objective response has been translated and used in clinical and drug approval procedures [49]. For particular tumor-therapy combinations, research studies have sought to optimize the definition of objective response. To assess the predictive power of various biomarker iterations for important clinical endpoints, comparisons between them can be made. IBs have a critical role in this, as shown in Figure 3 [51].

IBs have a lot of potential for cancer research and oncology practice, but in order to fully realize that potential, they must go through validation and qualification processes.

### 3.3. Needle Biopsy

Imaging tests are essential in identifying and tracking cancer [52]. These examinations use various forms of energy, such as X-rays, sound waves, radioactive particles, or magnetic fields, to produce finely detailed images that reveal important details about the structure and location of the tumor [53,54,55,56]. It is crucial to remember that imaging tests do have their limitations. They cannot identify specific cancer cells, and their results are inconclusive. Imaging tests are typically validated by biopsy [57].

**Figure 4:**
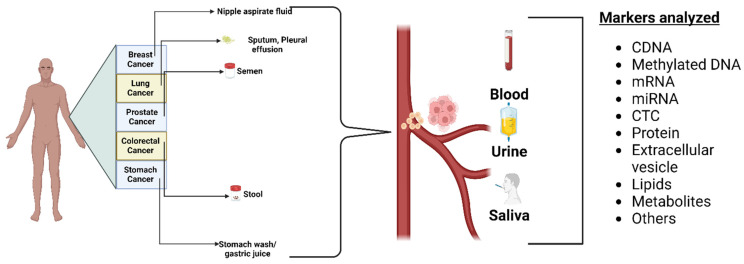
Commonly used non-invasive techniques for examining biomarkers in solid tumors.

A cancer biopsy is a test for diagnosis employed to identify the kind and properties of the tumor cells and confirm or rule out the existence of cancer. Findings are crucial for making additional medical choices (grading of tumor; chemotherapy vs. radiation vs. immunotherapy) [58,59,60,61]. In accordance with the precise spot and accessibility of the suspicious region, biopsies can be carried out using a variety of approaches (Figure 4), including surgical biopsies, endoscopic biopsies, and needle biopsies [62]. Needle biopsy may employ a larger needle for collecting large tissue specimens or a fine needle aspiration for gathering a small sample from cells and fluid. A special needle with a suction mechanism is used in vacuum-assisted biopsy for acquiring tissue specimens. These methods provide versatility in gathering appropriate samples for analysis [63,64]. A non-surgical procedure called a core needle biopsy is used to collect tissue samples for evaluation. Ultrasound- or vacuum-assisted biopsy approaches may be utilized in hard-to-reach places [65].

### 3.4. Tissue Imaging

Immunohistochemistry (IHC), an approach used for tissue image processing, allows researchers to analyze particular proteins or antigens in tissue samples. Antibodies designed to bind to specific protein targets in tissue sections are used in this procedure. A secondary antibody is coupled to a recognition molecule after the primary antibody is bound to its target. The results are visualized under a microscope. The results are typically in the form of an alteration in color or fluorescence, indicating the target protein’s existence and location. IHC is a widely used method in pathology studies and diagnostics that can reveal important details about the arrangement, expression levels, and localization of particular proteins in tissue specimens.

There is a growing need for diagnostic methods that can identify cancer early using functional and morphological data. Terahertz (THz) and infrared radiation-based imaging methods (FTIR and Raman) are two examples of contemporary medical imaging systems that are currently being researched and validated. Non-ionizing, non-invasive, label-free detection of cancer is possible with THz imaging. THz and other spectroscopic-based imaging are pursued to identify cancer margins during surgeries [1]. THz waves are highly sensitive to alterations in tissue water content, making it possible to monitor hydration levels. THz technology can track DNA’s molecular resonance, providing a chance to look into DNA methylation as a potential cancer biomarker [65]. For use in clinical and translational cancer diagnosis, contrast agents may also improve THz imaging.

Different kinds of spectroscopies are also being used for tumor imaging and cancer detection. Because cervical cancer is a common condition with a gradual onset, early and precise identification is essential for better patient outcomes. One study collected Raman spectral data from 233 cervical cancer patients and proposed a 1D hierarchical convolutional neural network (H-CNN) that combines deep learning in Raman spectroscopy with prior knowledge of hierarchical classification relations [66]. The results of the experiments show that H-CNN performs better than conventional methods in terms of accuracy, stability, and sensitivity when it comes to identifying tissue sections [66].

## 4. Types of Cancer Biomarkers

### 4.1. Genetic Biomarkers

#### 4.1.1. Mutations and Gene Alterations

Mutations and gene alterations are important cancer biomarkers that can provide valuable information about the underlying genetic changes driving the development and progression of cancer. Here are some examples of mutation- and gene alteration-based cancer biomarkers. The BRAF V600E mutation activates cell growth, aiding targeted therapy selection in melanoma patients [67]. EGFR mutations (e.g., exon 19 deletions, L858R point mutation) increase sensitivity to EGFR inhibitors in non-small-cell lung cancer (NSCLC) [68]. In colorectal, KRAS mutations (30–40% cases) activate signaling pathways, affecting treatment response [69]. BRCA1/BRCA2 mutations increase cancer risk in breast/ovarian cancer, and guide therapy selection [70]. HER2 amplification/overexpression indicates aggressive behavior in breast/gastric cancer, and is generally treated with anti-HER2 antibodies [71]. IDH mutations affect cellular metabolism and serve as diagnostic and prognostic markers in glioma patients [72].

#### 4.1.2. Gene Expression Profiles

Gene-expression-profile-based cancer biomarkers involve analyzing the patterns of gene expression in cancer cells to provide insights into tumor behavior, prognosis, and treatment response. Here are some examples of gene-expression-profile-based cancer biomarkers:

Oncotype DX in Breast Cancer: Oncotype DX is a genomic test that assesses the expression of a panel of about 16 genes involved in breast cancer. It provides a recurrence score (RS) that predicts the likelihood of disease recurrence and guides treatment decisions, particularly in early-stage hormone receptor-positive breast cancer. The genes in question are ERBB2 (also known as HER2), ESR1 (estrogen receptor 1), PGR (progesterone receptor), BIRC5 (survivin), SCUBE2 (signal peptide, CUB domain, EGF-like 2), STK15 (Aurora kinase A), BCL2 (B-cell lymphoma 2), MKI67 (Ki-67), GSTM1 (glutathione S-transferase mu 1), CD68 (cluster of differentiation 68), BAG1 (BCL2-associated athanogene 1), MMP11 (matrix metallopeptidase 11), CTSL2 (cathepsin L2), GRB7 (growth factor receptor-bound protein 7), GSTM1 (glutathione S-transferase mu 1), and CDKN1B (cyclin-dependent kinase inhibitor 1B) [73].

MammaPrint in Breast Cancer: MammaPrint is a gene-expression-based assay used to analyze the activity of a set of genes (~18 genes) in breast cancer. It provides a genomic risk score (RS) that helps determine the risk of distant metastasis and assists in treatment decision making, particularly in early-stage breast cancer. The list of genes includes AURKA (Aurora kinase A), BIRC5 (survivin), CCNB1 (cyclin B1), CDC2 (cell division cycle 2), CKS1B (CDC28 protein kinase regulatory subunit 1B), DLG7 (discs large homolog 7), ERBB2 (also known as HER2), ESR1 (estrogen receptor 1), FOXM1 (forkhead box M1), MMP11 (matrix metallopeptidase 11), MYBL2 (myb-related protein B), NDC80 (kinetochore protein NDC80 homolog), NEK2 (NIMA-related kinase 2), RACGAP1 (Rac GTPase-activating protein 1), RRM2 (ribonucleotide reductase M2 subunit), STK15 (Aurora kinase A), TYMS (thymidylate synthase), and UBE2C (ubiquitin-conjugating enzyme E2C) [74].

Prosigna in Breast Cancer: Prosigna, also known as PAM50, is a gene expression assay that classifies breast cancer into distinct subtypes based on the expression levels of a set of 50 genes. This classification helps predict prognosis and response to hormone therapy, aiding in treatment planning for breast cancer patients. The following 28 genes are included: ACTR3B, BAG1, BCL2, BIRC5, CCNB1, CCNE1, CDC20, CENPF, CEP55, DSCC1, EGFR, ERBB2 (HER2), ESR1, FOXA1, GRB7, KRT14, KRT17, KRT5, MKI67 (Ki-67), MELK, NDC80, PGR, RBBP8, RRM2, SFRP1, SFRP4, SFRP5, and THSD7A [75].

Decipher in Prostate Cancer: Decipher is a genomic test for prostate cancer that evaluates the gene expression profile of a tumor. It provides a genomic risk score (GRS) that predicts the likelihood of disease recurrence after prostate surgery and helps guide decisions regarding adjuvant therapy. The genes in question are ACTB (actin beta), ANLN (anillin, actin binding protein), AURKA (aurora kinase A), AURKB (aurora kinase B), BIRC5 (survivin), CCNB1 (cyclin B1), CDCA3 (cell division cycle-associated 3), CDCA8 (cell division cycle-associated 8), CDC20 (cell division cycle 20), CDC45L (cell division cycle 45 like), CDC6 (cell division cycle 6), CDC7 (cell division cycle 7), CDK1 (cyclin-dependent kinase 1), CHEK1 (checkpoint kinase 1), CHEK2 (checkpoint kinase 2), CNTNAP3B (contactin-associated protein 3B), HMMR (hyaluronan-mediated motility receptor), KIF20A (kinesin family member 20A), KIF2C (kinesin family member 2C), MELK (maternal embryonic leucine zipper kinase), MKI67 (Ki-67), NEK2 (NIMA-related kinase 2), NUSAP1 (nucleolar and spindle-associated protein 1), PTTG1 (pituitary tumor-transforming 1), RRM2 (ribonucleotide reductase M2 subunit), TOP2A (DNA topoisomerase II alpha), TPX2 (microtubule-associated protein), and UBE2C (ubiquitin-conjugating enzyme E2C) [76].

VeriStrat in Lung Cancer: VeriStrat is a blood-based protein signature test that measures the expression levels of specific proteins in the serum of lung cancer patients. It categorizes patients as either “VeriStrat Good” or “VeriStrat Poor,” indicating the likelihood of response to certain therapies, including EGFR tyrosine kinase inhibitors (TKIs). The specific genes associated with VeriStrat are proprietary information and not publicly disclosed. The test focuses on protein profiling rather than gene expression profiling. Some of the proteins are EGFR (epidermal growth factor receptor), VEGF (vascular endothelial growth factor), CRP (C-reactive protein), A1AT (alpha-1 antitrypsin), SAA (serum amyloid A), and ITIH4 (inter-alpha-trypsin inhibitor heavy chain 4) [77].

Gene Expression Classifier in Colon Cancer: A gene expression classifier, such as ColoPrint, is used to analyze the gene expression profile of colon cancer. It provides a molecular subtype classification that aids in determining prognosis and identifying patients who may benefit from chemotherapy, helping to guide treatment decisions. The listed genes include CDX2 (caudal type homeobox 2), GJA1 (gap junction alpha-1 protein), VIM (vimentin), SLC26A3 (solute carrier family 26 member 3), CDH17 (cadherin 17), CEACAM5 (carcinoembryonic antigen-related cell adhesion molecule 5), DSC2 (desmocollin 2), GUCA2B (guanylate cyclase activator 2B), FABP1 (fatty acid-binding protein 1), TFF3 (trefoil factor 3), GREB1 (growth regulation by estrogen in breast cancer 1), SATB2 (special AT-rich sequence-binding protein 2), CDX1 (caudal type homeobox 1), ZNF185 (zinc finger protein 185), MT1E (metallothionein 1E), ITGA1 (integrin subunit alpha 1), LGALS4 (galectin 4), IL8 (interleukin 8), LYZ (lysozyme), KLK11 (kallikrein-related peptidase 11), VIL1 (villin 1), S100P (S100 calcium-binding protein P), ANO1 (anoctamin 1), SLC4A4 (solute carrier family 4 member 4), and OLFM4 (olfactomedin 4). By assessing the activity levels of specific genes, these biomarkers help predict prognosis, guide treatment decisions, and identify patients who are likely to respond to particular therapies [78].

#### 4.1.3. DNA as a Cancer Biomarker

**Figure 5:**
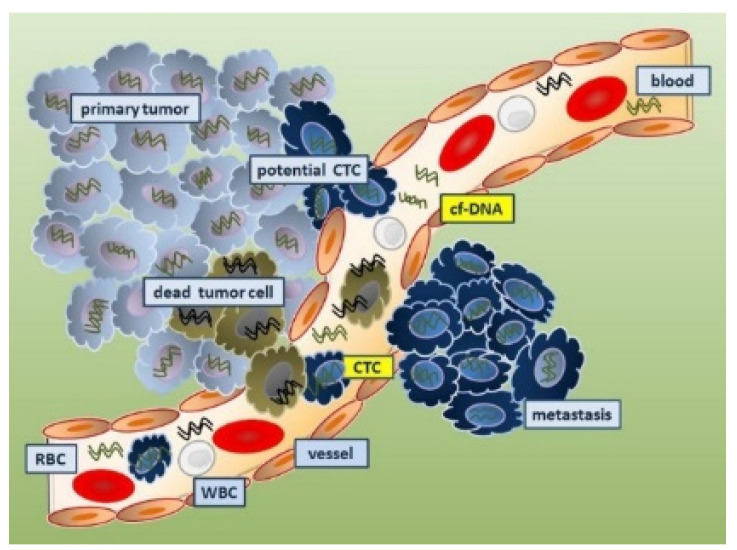
DNA from free cells and malignant cells in circulation. Circulating tumor cells (CTC) spread throughout the blood vessels after escaping from original locations and forming metastases in the distal organs. Dead cancer cells or expanding tumor cells release cell-free DNAs (cf-DNAs) into the bloodstream. RBC = red blood cell; WBC = white blood cell [88].

**Figure 6:**
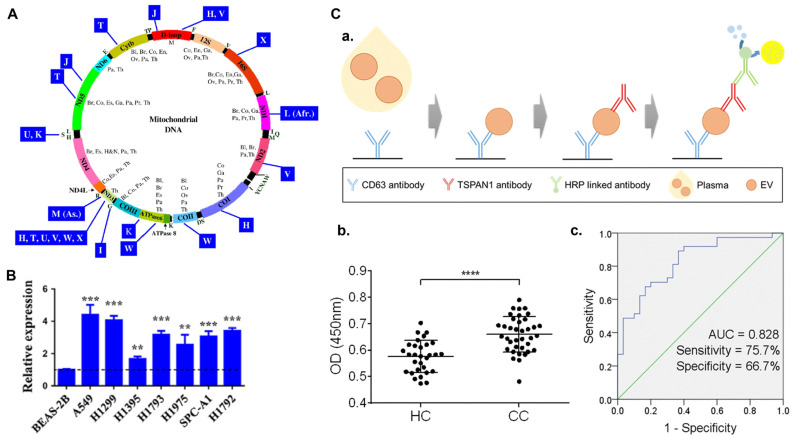
(**A**) Genes and haplogroups are depicted on a plot of the mitochondrial genome. Genes are depicted within the circle, whereas haplogroups are depicted outside. The following acronyms are provided inside the circle for tumors where alterations in mitochondrial genome have been mentioned: Co, colon cancer; H&N, head and neck cancer; Pa, pancreatic cancer; Ov, ovarian cancer; Br, breast cancer; Th, thyroid cancer; Bl, bladder cancer [79]. (**B**) Graphical representations of Hsa circ 0013958 levels in seven lung adenocarcinoma (LAC) cell lines which were analyzed using PCR [109]. The normalization was done with respect to BEAS-2B cell line. Here, ** *p* < 0.01 and *** *p* < 0.001. (**C**) Schematic representation of the diagnostic performance of TSPAN1-positive extracellular vesicles in plasma. (**a**) The encapsulated anti-CD63 antibody was utilized to trap TSPAN1-positive small extracellular vesicles in plasma, and the anti-TSPAN1 antibody was applied to detect them. (**b**) In the plasma of healthy controls (HC, *n* = 30) and colon cancer patients (CC, *n* = 37) TSPAN1-positive small extracellular vesicles were detected. The Mann–Whitney test was performed to determine significance. **** *p* < 0.0001. (**c**) The ROC curves for distinguishing between healthy controls (HC) and colorectal cancer patients were evaluated. The TSPAN1’s AUC, specificity, and sensitivity are presented [111].

The initial markers tested for tumor staging were circulating DNA, as shown in Figure 5. Elevated concentrations of serum DNA have been linked to cancer (most particularly, metastatic cancer). Oncogene alterations, mismatch-repair gene mutations, and mutations in tumor suppressor genes can all be used as DNA biomarkers. In over 50% of sporadic malignancies, mutations in the p53 tumor suppressor gene are found, and mutations in the KRAS oncogene indicate metastatic spread [67,68,69]. A TP53 mutation passed down through the generations (Li–Fraumeni syndrome) raises the likelihood of acquiring several of the same malignancies. Several genes have single nucleotide polymorphisms, including RAD1, CYP1A1, and BRCA1/2 (breast cancer), PGS2 (lung cancer), and XRCC1, p53, and ATM (lung, head, and neck cancers). Diagnosis has been associated with mutations in DNA nucleotides in tumor promoters such as APC, RAS, and tumor suppressor genes. Tissue, sputum, serum, saliva, cerebrospinal fluid (CSF), bronchial tear, tumor cells circulating in the bone marrow, and blood are all potential sources of DNA [79,80,81]. Mutations in mitochondrial DNA have been postulated as diagnostics biomarkers for various malignancies [79,82,83]. Haplotype analysis was used to investigate the mitochondrial inheritance pattern in cancer patients. Researchers used polymerase chain reaction to look for critical polymorphic locations in the mitochondrial DNA in specimens from cancer patients and healthy subjects to see if there is a link connecting mitochondrial genotype and cancer. Nine mitochondrial genomic haplogroups have been described, namely, H, I, J, K, T, U, V, W, and X. U is linked with a high chance of developing renal and prostate cancer among these haplogroups [79]. In Figure 6A, tumor DNA is discharged into the bloodstream. Employing circulating DNA allows for a less invasive approach and simpler sequential tracking. The DNA levels can range from 0.01 to 90% of total DNA in the blood, depending on parameters including tumor location, tumor burden, the amount of tumor necrosis, tumor cell turnover, and accessibility to the vasculature [84]. Circulating DNA-based biomarkers for cancers may even be more effective in detecting the genetic changes which induce acquired resistance to specific treatments, and are more precise than conventional biomarkers, reducing false-positive incidences. As opposed to circulating protein biomarkers, circulating DNA biomarkers are advantageous as they have a shorter half-life (2.5 h) and a broader dynamic range, but they need appropriate specimen collection time [84,85]. Certain tumor-related genetic changes, including those in BRAF, KRAS EGFR, KIT, ALK, HER2, and PDGFR, were discovered using ctDNA-based assays [86]. Spindler et al. reported the levels of expression of plasma KRAS mutant alleles of colorectal cancer patients [87].

#### 4.1.4. RNA as a Cancer Biomarker

Differential display, RT-qPCR, bead-based approaches, and micro-array analysis are among the techniques applied to diagnose potential biomarkers at the RNA expression level [89]. MicroRNAs (miRNAs) are short non-coding RNAs linked to clinical features in several cancers. The expression of particular miRNA populations is related to clinical features in a time- and tissue-dependent pattern [90,91,92]. To promote tumorigenesis, metastasis, immune evasion, and angiogenesis, microRNAs regulate the transcription of their target mRNAs [93,94]. Tumor microRNA profiles can be used to identify important subgroups, survival rates, and responsiveness to therapy.

Furthermore, cancer-associated microRNA markers may be detectable in bodily fluid, enabling individuals with cancer microRNAs to be monitored with less invasive approaches [95]. In 2002, the first report on microRNA dysregulation in cancer was published. In chronic lymphocytic leukemia, groups of two microRNAs (miR-16 and miR 15) were discovered [96]. In another study [97], when compared with healthy controls, the miRNA-483-3p expression was reported to be substantially greater in pancreatic ductal adenocarcinoma (*p* < 0.01). The plasma miRNA-483-3p expression was greater in intraductal papillary mucinous neoplasm, and miRNA-21 expression was correlated with metastases to liver and lymph nodes (*p* < 0.01) [97]. miRNAs control a variety of targets, serving as either tumor suppressors or oncogenes. The proliferation, invasion, and migration of colorectal cancer cells are inhibited by miR-18a [98], miR-205-5p [99,100], and miR-155 [101,102], while miR494 [103], miR-17-3p [104], and miR-598 [105] stimulate proliferation and migration.

Circular RNAs, which are non-coding RNAs with a closed loop structure, are generated by the splicing of a precursor RNA (pre-mRNA) and covalent binding of 3′ poly(A) tails and 5′capping [106] CircRNAs play a significant role in gene regulation [107,108]. According to Zhu et al. [109], Hsa circ 0013958 was higher in all lung adenocarcinomas, with 20 circRNAs down-regulated and 39 up-regulated. The study reported that Hsa circ 0013958 might be applied as a potent non-invasive marker for the early diagnosis of lung adenocarcinoma. To find Hsa circ 0013958, researchers used real-time PCR to look for its levels in lung adenocarcinoma (LAC). Compared to the healthy human bronchus epithelial cell line, Hsa circ 0013958 levels were reported to be higher in LAC cell lines (Figure 6B) [109]. According to Song et al. [110], the level of expression of Hsa circRNA 101996 in cervical cancer was linked positively with tumor size, TNM staging, and lymphovascular invasion. Further, the upregulation of Hsa circRNA 101996 are linked to poor prognosis. They discovered that miR-8075, which is regulated by Hsa circRNA 101996, inhibits TPX2 upregulation and promotes the proliferation and metastasis of cervical cancer.

#### 4.1.5. Epigenetics as a Cancer Biomarker

Epigenetic alterations are potent biomarkers for cancer as they are frequent for specific genes, are stable, and can be detected in a minimally invasive mode. Numerous studies have discovered that DNA methyltransferases that insert methyl groups into cytosine groups of DNA are changed in cancer cells [112]. The hypermethylation of local CpG island promoter silences the tumor suppressor genes, stimulating their gene mutations. NKX2-6, SPAG6, PER1, and ITIH5 gene methylation was detected in breast cancer patients’ serum [113]. The hypermethylation of promoter p16 in serum DNA, for instance, is linked to recurring colorectal cancer. The methylation of the RASSF1A and p16Ink4 genes has been related to a 15-fold elevation in the comparative risk of lung cancer. The methylation status of multiple genes in clinical specimens might be a viable non-invasive technique for detecting smokers at risk of developing lung cancer [114]. DNA promoter hypermethylation of the RASSFIA or BRCA1 gene was found in 68% of ovarian tumor tissue, according to Ibanez de Caceres et al. [115]. Through the activation of oncogenes and the inhibition of tumor suppressor genes, histone-acetylation plays a dual role in tumor genesis and progression. Cang et al. [116] indicated that the degree of acetylation of histone H3 at specific areas in prostate cancer cell lines is lower compared to healthy tissue specimens, followed by enhanced histone deacetylase activity. A comparison of several genetic biomarkers is represented in Table 1.

### 4.2. Protein Biomarkers

#### Proteins as Cancer Biomarkers

**Figure 7:**
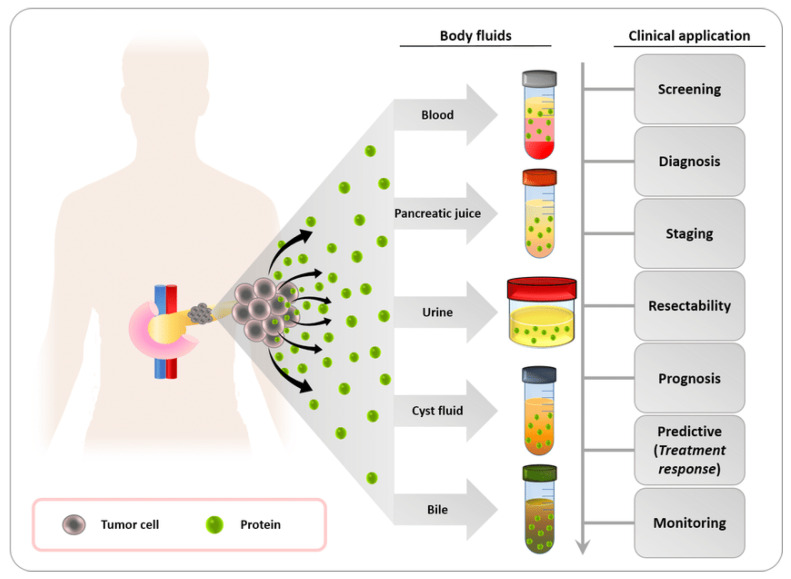
Identification of possible protein biomarkers for pancreatic cancer using bodily fluids. Bodily fluids that include cancer-derived proteins include bile, blood, pancreatic juice, urine, and pancreatic cyst fluid. For the management of pancreatic cancer patients, these proteins have a high potential as tumor biomarkers and a variety of clinical applications, including screening in high-risk populations for pancreatic cancer, early diagnosis, disease staging, the evaluation of tumor resection and prognosis, the prediction of therapy response to inform treatment decisions, and real-time patient monitoring [133].

The proteome is a complex system made up of several proteins which interact with one another in dynamic intermolecular interactions and posttranslational alterations. Because they modulate molecular processes and pathways in normal and cancerous cells, proteomic markers are relevant to tumorigenesis and progression [131,132]. Proteins from pancreatic cancer can be found in a number of bodily fluids, including bile, pancreatic juice, urine, and fluid from pancreatic cysts, as shown in Figure 7. These proteins have a great deal of potential as useful biomarkers with a range of therapeutic applications, including early identification, illness staging, treatment prognosis, and in-flight patient monitoring. The majority of the FDA-approved cancer biomarkers in clinical usage are single proteins obtained from serum. HCG, AFP, and LDH are utilized to stage testicular cancer. For instance, the expression of HER2/NEU and cytokeratins can be applied to improve breast cancer prognosis.

The biomarkers CD171, CD151, and tetraspanin 8 were the most significant indicators between lung cancer patients of all subgroups and healthy individuals [134]. Recent research reveals novel plasma biomarker proteins that may aid in the early diagnosis of bladder cancer. The amount of haptoglobin was found to be significantly higher in patients with low-grade bladder cancer, suggesting that this protein may have a role in the initial stages of bladder tumorigenesis. With reasonable specificity and sensitivity (AUC > 0.87), haptoglobin could differentiate between patients with low-grade bladder cancer and controls [135]. Figure 6C demonstrates the verification of TSPAN1 by ELISA in small extracellular vesicles from colorectal cancer patients (*n* = 37) and healthy controls (*n* = 30). Figure 6C(a) shows TSPAN1 was trapped by utilizing a coated anti-CD63 antibody and detected using an anti-TSPAN1 antibody. The TSPAN1 levels were more significant in CC patients than in HCs (healthy controls) (Figure 6C(b)). Receiver operating curves (ROC) were created using ELISA findings to demonstrate the diagnostic performance (Figure 6C(c)). The area under the curve (AUC) for TSPAN1 was calculated. TSPAN1 had an AUC of 0.828 for differentiating between healthy controls and colorectal cancer patients, with a specificity of 66.7% and a sensitivity of 75.7%. TSPAN1 appears to be a helpful non-invasive biomarker for colorectal cancer diagnosis, based on these findings [111]. Other common protein biomarkers for different cancer diagnoses are listed in Table 1. For high-throughput profiling with microgram levels of protein, technologies such as surface plasmon resonance (SPR), two-dimensional polyacrylamide gel electrophoresis (2D-PAGE), differential in-gel electrophoresis (DIGE) [103], and multidimensional protein identification technology (MudPIT) can be utilized. Table 2 features examples of protein tumor markers, along with their typical concentrations in the healthy population and in cancer patients. It should be noted that the concentrations provided are approximate and can vary depending on factors such as the specific assay used and individual variations.

Overexpressed or mutated proteins: Cancer biomarkers include mutated or overexpressed proteins with varying concentration levels depending on cancer type, stage, and individual characteristics. Common examples are HER2 in breast and gastric cancers, EGFR in lung, colorectal, and head and neck cancers, KRAS in colorectal, pancreatic, and lung adenocarcinoma, BRAF in melanoma and colorectal cancer, ALK in some NSCLC cases, and PSA as a prostate cancer biomarker. Detection methods such as IHC, FISH, PCR, and NGS are utilized for assessment. These biomarkers play a crucial role in cancer diagnosis, classification, and treatment decision making.

Signaling pathways and protein interactions: Signaling pathways and protein interactions play a critical role in cancer development and progression. The dystregulation of these pathways and interactions can lead to uncontrolled cell growth, invasion, and metastasis. Several key signaling pathways have been implicated in cancer, including the PI3K/AKT/mTOR pathway, Wnt/β-catenin pathway, Ras/Raf/MEK/ERK pathway, Notch signaling pathway, and TGF-β signaling pathway. The activation of the PI3K/AKT/mTOR pathway promotes cell survival, proliferation, and resistance to apoptosis. The Wnt/β-catenin pathway, when aberrantly activated, leads to altered gene expression, promoting cell proliferation and tumor progression. Mutations in Ras genes and dysregulation of downstream components in the Ras/Raf/MEK/ERK pathway commonly occur in cancers, resulting in uncontrolled cell growth, survival, and metastasis. The dysregulation of the Notch signaling pathway can drive tumor cell proliferation, survival, and angiogenesis. The TGF-β signaling pathway, with its diverse roles in normal development and cancer, when dysregulated, contributes to cancer progression, including increased cell proliferation, epithelial-to-mesenchymal transition (EMT), and immune evasion.

In addition to signaling pathways, protein interactions also play a significant role in cancer. For example, in HER2 signaling, HER2 forms complexes with other receptors like EGFR and HER3, leading to the downstream activation of signaling cascades such as the PI3K/AKT and MAPK pathways. These interactions promote cell growth and survival, contributing to cancer progression. Furthermore, interactions between immune checkpoint proteins, such as programmed cell death protein 1 (PD-1) and its ligand PD-L1, have been observed in cancers. These interactions can suppress the immune system, enabling tumor immune evasion and facilitating cancer growth. Understanding these signaling pathways and protein interactions provides valuable insights into the mechanisms underlying cancer development and guides the development of targeted therapies aimed at disrupting these pathways and interactions to inhibit tumor growth and improve patient outcomes. Table 3 provides a thorough list of cancer types together with the associated immunotherapies that have been approved by the FDA.

### 4.3. Metabolic Biomarkers

#### 4.3.1. Metabolites and Metabolic Pathways

Metabolites and metabolic pathways are essential in cancer cells as they undergo alterations to support their growth and survival [155]. Metabolic biomarkers derived from these pathways and metabolites can provide valuable information about cancer metabolism and aid in diagnosis, prognosis, and treatment. Here are some examples of metabolites and metabolic pathways used as metabolic biomarkers in cancer:

Glycolysis: Increased glucose consumption and aerobic glycolysis (the Warburg effect) are characteristic metabolic changes in cancer cells. Biomarkers associated with glycolysis include the following: (1) Lactate: elevated lactate levels in tumor tissues or serum indicate increased glycolytic activity. (2) Glucose transporters (e.g., GLUT1): the overexpression of glucose transporters facilitates glucose uptake in cancer cells [156].

TCA Cycle (Citric Acid Cycle): The tricarboxylic acid (TCA) cycle plays a vital role in energy production and biosynthesis [157]. The dysregulation of the TCA cycle intermediates can serve as metabolic biomarkers: (1) Fumarate and succinate: the accumulation of fumarate and succinate is associated with specific genetic mutations, such as in fumarate hydratase (FH) and succinate dehydrogenase (SDH), respectively. (2) α-Ketoglutarate: altered α-ketoglutarate levels are observed in certain cancer types, such as renal cell carcinoma [158].

Lipid Metabolism: Altered lipid metabolism is common in cancer cells, and several metabolites and pathways are associated with lipid metabolism biomarkers: (1) Choline: increased choline levels, measured using magnetic resonance spectroscopy (MRS), are found in several cancers, including breast and prostate cancers. (2) Fatty acid synthase (FASN): the overexpression of FASN, an enzyme involved in fatty acid synthesis, is observed in various cancers [159,160].

Amino Acid Metabolism: Cancer cells exhibit altered amino acid metabolism, resulting in the production and consumption of specific metabolites: (1) Glutamine: increased glutamine uptake and utilization are common in cancer cells. Glutamine metabolism is associated with pathways such as the TCA cycle and nucleotide synthesis. (2) Serine and glycine: dysregulated serine and glycine metabolism is observed in several cancers, including breast and colorectal cancers [161].

Nucleotide Metabolism: Rapidly dividing cancer cells require nucleotides for DNA and RNA synthesis. Biomarkers related to nucleotide metabolism include deoxythymidine (dThd). Elevated levels of dThd have been associated with certain cancer types and can be detected in urine or plasma [162].

These are examples of metabolites and metabolic pathways used as metabolic biomarkers in cancer. By analyzing these biomarkers, researchers and clinicians can gain insights into the metabolic alterations specific to cancer cells and develop targeted therapies aimed at disrupting cancer metabolism.

#### 4.3.2. Metabolic Imaging Techniques

Metabolic imaging techniques are used to visualize and assess the metabolic activity of cancer cells. These techniques provide valuable information about tumor metabolism and can aid in cancer diagnosis, staging, treatment planning, and monitoring. Here are some commonly used metabolic imaging techniques in cancer:

Positron Emission Tomography (PET): PET imaging utilizes radiolabeled tracers that are taken up by cells based on their metabolic activity. The most commonly used tracer in PET imaging is fluorodeoxyglucose (FDG), a glucose analog. FDG-PET measures glucose metabolism and is particularly useful in detecting and staging various cancers, including lung, colorectal, and breast cancers [163].

Magnetic Resonance Spectroscopy (MRS): MRS allows the non-invasive assessment of metabolite concentrations in tissues. It provides information on metabolites such as choline, creatine, and lactate, which are associated with cellular metabolism. MRS is used in brain tumor imaging to assess tumor grade, identify tumor margins, and monitor treatment response [164].

Magnetic Resonance Imaging with Hyperpolarized Substrates (HP-MRI): HP-MRI is an emerging technique that utilizes hyperpolarized substrates, such as pyruvate or fumarate, which are metabolized in real time to visualize metabolic pathways. This technique provides dynamic information on metabolic fluxes, such as glycolysis or TCA cycle activity, and holds promise for assessing tumor metabolism and treatment response [165].

Single-Photon Emission Computed Tomography (SPECT): SPECT imaging uses radiotracers that emit gamma rays to detect specific metabolic processes. SPECT can be used to assess various metabolic functions, such as blood flow, metabolism, and receptor binding. Examples include technetium-99m sestamibi for imaging myocardial perfusion and iodine-123 ioflupane for imaging dopamine transporter function in neuroendocrine tumors [166].

Dynamic Contrast-Enhanced Magnetic Resonance Imaging (DCE-MRI): DCE-MRI involves the administration of a contrast agent to evaluate the tumor’s vascularity and blood flow. By measuring the kinetics of contrast agent uptake and washout, DCE-MRI provides information on tumor perfusion, angiogenesis, and vascular permeability. It is used in various cancers, including breast, prostate, and brain tumors [167].

Optical Imaging: Optical imaging techniques, such as fluorescence imaging and bioluminescence imaging, can be used to assess metabolic processes at a cellular level. Fluorescent probes and reporter genes are utilized to visualize specific metabolic activities, such as pH, reactive oxygen species, or enzyme activity. Optical imaging is commonly employed in preclinical research and experimental studies. These metabolic imaging techniques offer complementary information about tumor metabolism and aid in understanding the biological characteristics of cancer cells. By providing functional and metabolic data, these techniques assist in personalized treatment planning, monitoring treatment response, and guiding therapeutic interventions.

#### 4.3.3. Molecular Probes and Contrast Agents

Molecular probes and contrast agents are invaluable tools in cancer research and clinical imaging. They are designed to specifically target and highlight certain molecular features or physiological processes associated with cancer. Here are some examples of molecular probes and contrast agents used in cancer:

Fluorescent Probes: Fluorescent probes emit light at specific wavelengths when excited by the light of a different wavelength. They can be conjugated to antibodies or other targeting molecules to visualize specific cancer-related targets or processes. For example, fluorescently labeled antibodies can be used to target and detect specific proteins or receptors overexpressed in cancer cells [168].

Magnetic Resonance Imaging (MRI) Contrast Agents: MRI contrast agents enhance the contrast between normal and cancerous tissues in MRI scans. These agents often contain gadolinium, manganese, or iron oxide nanoparticles. They can help visualize tumor morphology, angiogenesis, and tissue perfusion. Examples include gadolinium-based contrast agents and superparamagnetic iron oxide nanoparticles (SPIONs) [169].

Positron Emission Tomography (PET) Tracers: PET tracers are radiolabeled molecules that are administered to patients and emit positrons, which can be detected by PET scanners. They are designed to target specific molecular pathways or processes associated with cancer. For example, fluorodeoxyglucose (FDG) is a radiolabeled glucose analog used to detect increased glucose metabolism in cancer cells, and 18F-fluorothymidine (FLT) is used to assess cell proliferation by targeting DNA synthesis [170].

Ultrasound Contrast Agents: Ultrasound contrast agents are microbubbles filled with gas that enhance the contrast during ultrasound imaging. These agents can help visualize blood flow, angiogenesis, and tumor vascularity. Microbubbles can be conjugated with targeting ligands to selectively bind to specific markers on cancer cells or blood vessels [171].

Near-Infrared (NIR) Imaging Probes: NIR imaging probes emit light in the near-infrared spectrum, which can penetrate deeper into tissues. They are used for the non-invasive imaging of tumors, lymph nodes, and other structures. NIR probes can target specific cancer markers or processes, allowing for real-time imaging during surgery or molecular imaging studies [172].

### 4.4. Cells as Cancer Biomarkers

Cells tend to emerge in circulation in advanced stages of tumors, where they can be readily tracked. Modern clinical practices have successfully exploited cancer and immune cells as a promising biomarker for the prognosis of specific malignancies, while its relevance in other tumors is still being studied.

#### 4.4.1. Circulating Tumor Cells as Cancer Biomarkers

In the realm of cancer, circulating tumor cells (CTCs) are basic yet effective biomarkers. The existence of CTCs has been demonstrated to determine patient survival with invasive breast cancer at various periods during treatment [173]. Cancer treatment targets (CTTs) are better predictors of prognosis than traditional tumor markers (e.g., CA27-29). The prevalence of therapeutic targets on CTCs can also influence the choice of an effective treatment regime, and the impact of treatment can be assessed after the initial cycle of medication [174]. The prevalence of CTCs has been reported to predict patient survival with metastatic breast cancer at various periods throughout treatment [173]. For patients undergoing systemic therapy for metastatic breast cancer, CTC gives an early and accurate indication of the progression of the disease and survival. CTC counts have been confirmed to be a consistent indicator for prognosis and therapy response in patients with metastatic prostate cancer. Schulze et al. reported that EpCAM-positive CTCs have a prognostic relevance to the detection of hepatocellular carcinoma [175]. CTC is an important prognostic marker in patients with metastatic breast cancer, prostate cancer, and lung cancer, according to a variety of clinical study results [176,177,178]. Various approaches are utilized for the molecular diagnosis of CTCs, including DNA sequencing, RNA sequencing, RNA in situ hybridization, and chromatin immunoprecipitation sequencing [179].

#### 4.4.2. Immune Cells as Cancer Biomarkers

**Figure 8:**
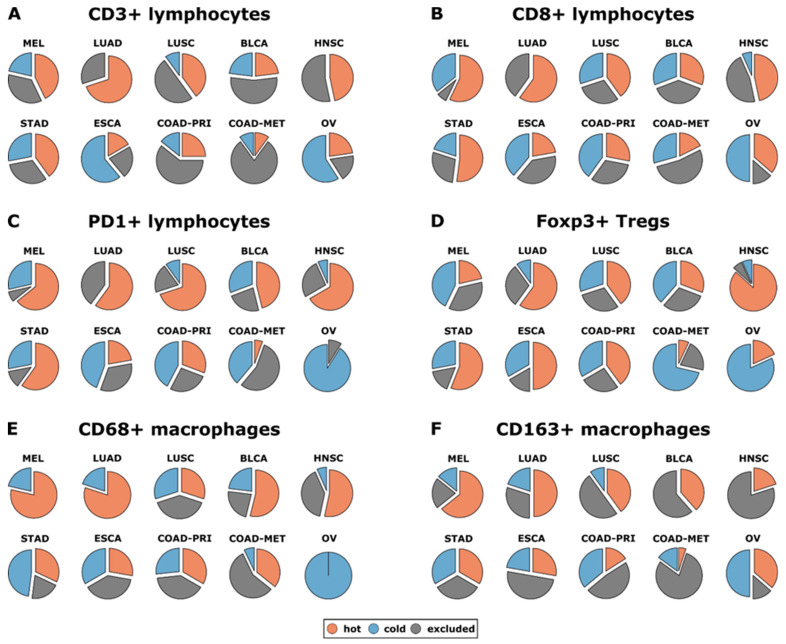
Distribution of different immune topological characteristics among various cancer types [186]. (**A**–**F**) Six distinct types of immune cells are analyzed in distinct tumor types such as lung squamous carcinoma (LUSC), lung adenocarcinoma (LUAD), melanoma (MEL), bladder (BCLA), stomach adenocarcinoma (STAD), head and neck squamous carcinoma (HNSC), esophageal squamous carcinoma (ESCA), colorectal liver metastasis (COAD-MET), colorectal primary (COAD-PRI), and ovarian (OV) cancer. All *n* = 965 tissue samples from *n* = 177 subjects were included in this analysis.

The immune system can differentiate between self-antigen and foreign antigens, promoting the maintenance of immune tolerance and inducing defensive immunity towards foreign antigens. Across several tumor entities, such as colorectal cancer and liver metastases, immune cell count in scanned tissue has already been employed to identify reliable and clinically useful biomarkers [180,181,182]. Macrophages and T lymphocytes are the tumor site’s most prevalent immune cells linked to clinical effects [183,184,185,186]. The histopathological examination of tumor-infiltrating lymphoid cells has been confirmed to be a credible and prognostically useful biomarker [187,188]. T cells aid in thwarting immune pathologies by sustaining self-tolerance [144,145]. Studies reported that upregulated regulatory T-cells (T-regs) expression had been linked to poor immunological responses to tumor antigens in cancer patients, indicating that it may promote immune dysregulation and tumor progression [189,190]. T-regs have already been detected in large numbers in patients with lung, breast, pancreatic, skin, and liver cancers, either in the bloodstream or in the tumor [189,191]. The prevalence of T-regs, which impair tumor-specific T-cell immunity, was negatively related to survival in ovarian cancer patients [192]. T-regs are essential for the emergence of metastasis to lungs in breast cancer, according to Olkhanud et al. [193]. The infiltration of T-regs in primary tumor sites has also been correlated with the prevalence of circulating tumor cell cells in breast cancer patients, implying involvement in cancer cell dissemination [194]. From a recent study, T-reg infiltration was found to be an independent marker of breast cancer survival (*p* = 0.01). Furthermore, patients with an infiltration of T-reg in distal metastases had a poor survival rate after recurrence (*p* = 0.039). In a recent study, Kather et al. [186] classified the tumors into three groups (based on the prevalence of immune cells inside and outside the tumor): “cold” (presence of fewer immune cells counts both outside and inside the tumor), “immune excluded (presence of fewer immune cells inside and more immune cells outside of the tumor), and “hot” (presence of more immune cells inside regardless of immune cell density outside). Additionally, they also measured the prevalence of different immune cells in various cancer types. They found that lung adenocarcinoma (LUAD), melanoma (MEL), lung squamous carcinoma (LUSC), and head and neck squamous carcinoma (HNSC) all had a high incidence of CD8-hot, CD3-hot, and PD1-hot tumors. Both primary colorectal cancer (COAD-PRI) and metastatic colorectal cancer (COAD-MET) exhibited a significantly higher percentage of CD3-excluded tumors (Figure 8). Figure 8 shows that over 50% of head and neck squamous carcinoma (HNSC), lung adenocarcinoma (LUAD), oesophageal cancer (ESCA), and stomach adenocarcinoma (STAD) specimens exhibited Foxp3-hot surface features, demonstrating that variations across cancer types were most apparent for Treg cells (Foxp3+). Additionally, although Foxp3-hot samples comprised 50% of all COAD-PRI specimens examined, Foxp3-cold samples contained the vast proportion of COAD-MET samples, as shown in Figure 8. This investigation suggests that immunological topographies can be utilized as biomarkers in patients suffering from solid malignancies [186].

#### 4.4.3. Cancer Stem Cells as Cancer Biomarkers

Within tumors, subpopulations of cancerous cells have long been identified that imitate the hierarchical developmental system of the healthy tissue from which cancer arises. The tumors are propelled and sustained by a small population of cells that can self-renew and produce the more differentiated cells that constitute the mass of the tumor [195]. Various researchers have termed the former subpopulation cancer stem cells (CSCs) to signify that exclusively these cells can produce new tumors when transplanted to animals with immune deficiency [196]. The cancer stem cell model has received a lot of attention recently. CSCs were first detected via research on acute myelogenous leukemia patients (AML). Numerous solid cancers, notably prostate cancer, glioblastoma, breast cancer, medulloblastoma, and melanoma, have been shown to contain CSCs [197]. Because CSC (cancer stem cell) destruction is expected to be a crucial factor in achieving cure, their prevalence has enormous consequences on both cancer biology and treatment. Self-renewal, tumor-originating capacity, asymmetric cell division, and differentiation capacity are all features that identify potential CSCs [198,199]. CD24, CD133, CD166 (ALCAM), CD44, EpCAM, CD29, Lgr5, ALDH1B1, and ALDH1A1 are some of the cytoplasmic and surface markers which have been utilized to detect putative cancer CSCs. Metastatic colon malignancies from patient populations were associated with an elevated expression of ALDH1B1 (*p* = 0.001) compared with healthy colon tissue [200]. Other investigations have correlated the degree of CD24 expression in colorectal tumors to lymphovascular invasion and decreased survival rates [201,202,203]. The expression of CD44v9 is associated with initial stage lung adenocarcinoma and epidermal growth factor receptor mutations in lung malignancies [204]. CD44 variants are also found in gastric malignancies, where they stimulate tumor initiation [205]. Thus, a CSC biomarker has been suggested as a marker for diagnosis, interventional, and prognostic purposes.

### 4.5. Lamins as Cancer Biomarkers

The nuclei of animal cells are identifiable by their well-defined chromatin compartmentalization and nuclear structure. In higher vertebrates, the intricate nuclear architecture has been associated with the surge in genomic intricacy and the demand for spatiotemporal control of gene expression. The nucleoplasm, nuclear pore complex, and lamina are the three main constituents of a standard multicellular nucleus. The lamina is a protein meshwork located on the inner nuclear membrane’s nucleoplasmic side. The main element of this lamina is a group of class V intermediate filaments proteins termed lamins which are abnormally expressed in tumors. Lamins control differentiation, apoptosis, gene expression, and DNA repair in a direct or indirect way. By analyzing abnormalities in the expression profile of lamins in different forms of malignancies, several researchers and cancer biologists were able to pinpoint the link between abnormal lamin expression and cancer subtype. The medication betulinic acid has anti-cancer properties in pancreatic cancer by limiting lamin B1 production, and it might be used as a biomarker for cancer. According to a report, it is linked to a more aggressive form of cancer and a worse prognosis for patients [206]. The research of lamin expression in testicular germ cell carcinoma might aid in the diagnosis of embryonic malignancy in tumors and serve as a prognostic biomarker. Cryo-preserved tissue slices of normal testis have been co-immunostained with both A- and B-type lamins to demonstrate differential expression, with just lamin C expressing in embryonic carcinoma, according to the study [207]. The expression of lamin A/C seems to be required for the progression of GBM tumors, and it may be associated with changes in the control of particular adhesion or invasion cellular pathways [208].

Scientists have looked into alterations in lamin patterns of expression in a variety of malignancy types in order to better understand the association between lamin transcription and cancer subgroups. Lamins, especially A-type lamins, communicate with transcription elements to control the growth and differentiation of cells [209]. In mature stem cells, the overexpression of the lamin A mutant inhibits the maturation and repair of tissue. The proliferation of cells is linked with decreased differentiation and zero or impaired gene expression for A-type lamins [210,211]. Lamins may function as indicators for cancer risk, forecasting the course and outcome of tumor growth. Nuclear lobulations and morphological alterations may result from lamin A depletion [212]. Colorectal malignancy, which has aberrant or misinterpreted lamin expression, is among the three most common malignancies worldwide. There is a strong correlation amongst lamin A/C expression, prognosis for patients, and the advancement of colorectal cancer, according to recent research. Death rates were almost twice as high in patients whose tumors tested positive for A-type lamin overexpression. Lamin A/C expression may serve as a risk signal for colorectal cancer-dependent mortality since it elevated T-plastin, reduced E-cadherin, and enhanced cell migration in colorectal cancer cells when GFP-lamin A was expressed ectopically. For a variety of gastrointestinal malignancies, appropriate lamin control is essential [213].

The expressions of lamin A/C, lamin B1, and lamin B receptor were analyzed in connection to the phases and clinical results of breast cancer. Reduced LMNB1 gene expression was connected to a worse outcome, while greater LMNA gene expression has been associated with initial cancer stages. It has been discovered that A-type lamins contribute to the development of breast cancer. Primary breast epithelial cells with lamin A/C expression knocked down by shRNA exhibit cancer-like shape and aneuploidy [214,215]. In the initial stages of human neurological tumors, there is frequently a reduction in lamin A/C, a protein that regulates neurogenesis. According to an investigation, cells with reduced lamin A/C levels showed resistance to drugs, migrated more frequently, and refused to complete differentiation. This means that although an increasingly invasive form of neuroblastoma may not promote differentiation, show enhanced migration, or show signs of therapy resistance, a lower lamin A/C expression may be a useful tool for detecting it [216,217].

Lamin A/C gives the nucleus morphological and mechanical integrity, which is essential for cell mobility, relocation, and infiltration in cancer cells. Table 4 below displays abnormal lamin expression and location in different types of cancer.

### 4.6. Galectins as Cancer Biomarkers

Galectins are a class of beta-galactoside-binding lectins widely found in all species. The genesis, progression, and pathological aggressiveness of tumors are linked to aberrant tumor-associated galectin expression. Rather than being a carcinoma diagnostic biomarker, galectin-3 is more of a malignancy function-related biomarker that can be applied in conjunction with certain other metabolic biomarkers. It is released into the tumor stroma and promotes tumor growth and angiogenesis [223]. Galectin-3 protein expression was much higher in breast tumor tissues relative to precancerous tissue, and triple-negative breast tumors have significantly higher levels of galectin-3 expression than other subtypes of breast cancer [224,225]. A study shows that serum galectin-3 levels in patients with metastatic prostate cancer were significantly greater than in healthy controls [226]. One type of cancer that shows higher expression of galectin-3 is pancreatic cancer. Pancreatic stellate cells (PSCs), which are cells that dwell in the pancreas, have been a focal point for research on the fibrosis linked to pancreatic cancer. The development of pancreatic cancer depends on the communication involving tumor cells and PSCs. Strongly detected in pancreatic tumors, galectin-3 stimulates PSCs via integrin signaling, promoting the development of malignancies and immune control [227]. In mice with tumor growth inhibition or blocking, tumor development is reduced and survival is increased. Gal-3 stimulates pancreatic cancer cell proliferation and invasion by interacting with Ras and turning on Ras signaling pathways, according to Song et al. [228].

Evidence from a variety of cancer types suggests that the expression of galectin-1 is frequently higher in tumor tissues in contrast with healthy or benign tissues. Malignancies of the reproductive organs, gastrointestinal tract, lymphatic malignancies, myeloproliferative tumors, respiratory and urinary system, thyroid, and skin tumors all exhibit this pattern [229,230,231,232,233,234,235,236,237,238,239,240]. Although three studies found that galectin-1 expression was decreased in head and neck squamous cell carcinoma, cancers of the uterus, and prostate cancer, these results do not agree with those of the majority of studies, which may indicate that patient demographics, tumor subtypes, or methodologies may differ [241,242,243].

The expression of galectin-7 varies between cancer types; it is expressed less in malignancies of the skin, cervix, and stomach and more in cancers of the gastrointestinal tract, breast, thyroid, larynx, and indolent lymphoproliferative diseases. The expression of galectin-7 is also dependent on the subtype of cancer and the location of the disease inside the cell; it is absent in carcinomas of basal cells and present in squamous cell tumors, which are head and neck malignancies [244,245,246,247,248].

Malignant tissues release circulating galectins, which can be utilized as a biomarker for diagnosis. There have been reports of elevated amounts of galectin-1 and -3 in thyroid, pulmonary, skin, bladder, colon, and breast cancers. However, they are not very useful in diagnosing thyroid cancer. Glycoproteins that bind to lectin may potentially function as diagnostic markers [249,250,251,252,253]. The circulating galectin-3 has predictive significance in individuals with stage III/IV melanoma, and raised levels of galectin-1 correspond with clinical progression in cases of Hodgkin lymphoma. Changes in blood galectin levels have been associated with pancreas, squamous cell tumors of the head and neck carcinoma, breast, and cancer of the gastrointestinal tract metastatic illness [254,255]. Table 5 below shows the abnormal expression of different types of serum galectins in particular types of cancer.

### 4.7. Carbohydrate Antigens as Cancer Biomarkers

Carbohydrate antigen (CA) biomarkers are cancer indicators that have been identified because of efforts to construct antibodies targeting extracts or cell lines derived from tumors. CA indicators are glycoproteins of high molecular weight. The most invariably utilized serum tumor biomarker for detecting malignancies of the digestive organs is CA19-9. The validated marker for detecting ovarian cancer recurrence and evaluating therapy response is CA-125 [304]. CA-125’s diagnosis sensitivity is limited, and it has been demonstrated that this glycoprotein is widely dispersed on the surface of cells in a variety of malignant or benign conditions other than ovarian cancer, leaving its efficacy in the diagnosis in jeopardy [115]. Carcinoembryonic antigen (CEA) is a glycoprotein found on the surface of cells that offers an important function in adhesion. CEA is produced by healthy mucosal cells, and its level in normal adults is as minimal as 2.5 ng/mL and as high as 5.0 ng/mL in people who smoke; but, in the existence of a tumor, it can reach 100 ng/mL. Increased CEA serum levels imply a higher risk of gastric, colorectal, breast, ovarian, and lung cancer [116]. CA125, also known as mucin16, is released by the serosal epithelium, with a typical level of 0–35 units/m [305]. CA-125 is useful for a variety of applications, including detection, prognostic, and post-treatment monitoring of disorders such as breast cancer, ovarian cancer, gastrointestinal carcinoma, and lymphoma [306,307,308].

### 4.8. Viruses as Cancer Biomarkers

Hepatocellular carcinoma (HCC) is among the most widespread viral-induced tumors [309]. Over 80% of HCC cases are reported in underdeveloped nations. The risk factors are chronic hepatitis viral infections, caused primarily by the prevalent hepatitis B virus (HBV), and hepatitis C virus (HCV) infection in a small percentage of HCC patients (12–17%) [309]. HBV can induce tumorigenesis by genomic instability mediated by its frequent incorporation in host DNA [310]. Cervical is the second most prevalent cancer in women, accounting for most cancer-related fatalities worldwide, and chronic infection with particular strains of HPV is the most prevalent trigger for cervical cancer. HPV has been detected in a substantial amount in anal, oral, penile, esophageal, vulvar, and vaginal cancers, as well as a tiny portion in laryngeal, lung, and stomach cancers in some regions of the world [311]. Cervical carcinoma samples were utilized to diagnose, clone, and sequence papillomaviruses for the first time. Antibodies to HPV (E6 and E7) produced by participants act as biomarkers of an HPV-related carcinoma [312]. Due to the sheer rise in HPV-related disease, especially HPV16 infection, oropharyngeal squamous cell carcinoma (OPSCC) is presumed to be the third most prevalent malignancy in middle-aged, non-Hispanic, white men by 2045. Hanna et al. [313] investigated antibody counts prior to and after post-chemotherapy medication; saliva samples from participants with HPV-positive OPSCC were evaluated against anti-HPV16 E7 and E6 IgG antibodies, and it was found that anti-HPV16 E7 IgG is higher than E6, and has a specificity of 100 percent and sensitivity of 71.4 percent. HPV-derived ctDNA was found in 56% of patients with oropharyngeal cancer (p16-positive) in a subsequent investigation. Following primary therapy, all the patients’ specimens were reported to be ctDNA-negative, and HPV-derived ctDNA was found at the stage of relapse, implying that HPV-derived ctDNAs can be used as a putative marker for detecting oropharyngeal cancer (p16-positive) relapse. The Epstein–Barr virus (EBV) was the earliest human virus linked to the development of cancer. It affects about 90% of people worldwide, with only a tiny fraction causing tumors [314]. An elevated risk for metastatic cancer was suggested by DNA of EBV in the peripheral blood [315]. Plasma EBV DNA identification and quantification is an effective diagnostic indicator for Hodgkin’s lymphoma and nasopharyngeal carcinoma detection, monitoring, and recurrence prediction [315,316]. As a result, viral biomarkers have prospects for application in diagnosing, staging, prognostic, and forecasting and evaluating therapeutic response. RNA viruses like human T-cell lymphotropic virus type 1 (HTLV-1) are an underlying cause for specific categories of leukemia [317].

### 4.9. Exosomes as a Cancer Biomarker

**Figure 9:**
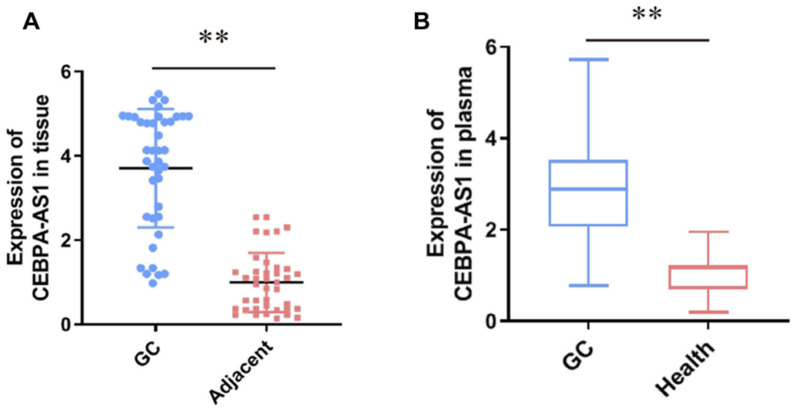
CEBPA-AS1 activity in human gastric cancer (GC). (**A**) CEBPA-AS1 activity in 40 patients’ GC tissues relative to paired neighboring tissues. (**B**) CEBPA-AS1 expression in plasma exosomes of GC patients versus healthy subjects. ** *p* < 0.01 [329].

Exosomes, which are the smallest (diameter of 30–150 nm) extracellular vesicles, are secreted by endothelial cells, erythrocytes, epithelial cells, dendritic cells, oligodendroglial cells, mesenchymal stem cells (MSCs), neural cells, and tumor cells [318,319]. Exosomes can serve as “cellular postmen” for carrying genomic material for inter- and intracellular communication because they are loaded with physiologically active components such as RNA, cytoplasmic proteins, cellular metabolites, and lipids [320]. Exosomes can be found in blood, breast milk, synovial fluid, amniotic fluid, urine, bronchoalveolar lavage fluid, pleural fluid, serum, and saliva [321,322]. Due to their widespread prevalence in physiological fluids and their resemblance to the contents of original cells, exosomes are potentially useful as circulating biomarkers for numerous kinds of cancers. By constructing or modulating the tumor microenvironment and encouraging angiogenesis and tumor invasion, tumor-derived exosomes (TEXs) serve a crucial role in tumorigenesis and progression [323,324]. TEXs contain a multitude of endogenous cargos which partly imitate the components and resemble the pathophysiological condition or signaling abnormalities of parent cells, rendering them potential biomarkers for early cancer detection. Exosomal proteins are emerging diagnosis and monitoring markers for cancers because there are plentiful cancer-related proteins in exosomes. In exosomes secreted from pancreatic cancer, overexpressed proteoglycan Glypican-1 (GPC-1) is found. Patients with pancreatic ductal adenocarcinoma (PDAC) were reported as having higher levels of exosomal protein 4 (CKAP 4) than healthy people. Exosomes containing CKAP 4 in the serum can be utilized as a potential biomarker for PDAC [325]. Trp5 (Transient Receptor Potential Channel 5) is overexpressed in exosomes from breast cancer, has a major function in drug resistance, and can be utilized to anticipate chemotherapy resistance in patients with breast cancer [326]. Exosomes have a double-layer lipid barrier that protects internal nucleic acids from being damaged. Consequently, exosomal nucleic acids can be potential indicators in cancer diagnostics. Hepatocellular carcinoma and other malignancies may benefit from exosomal miRNAs as potential serological markers [327]. Elevated exosomal miR-375 and miR-1290 levels in the plasma of prostate cancer (castration-resistant) patients were linked to a poor overall survival rate (OS) [328]. Piao et al. reported that exosomal long non-coding RNA (CEBPA-AS1) was detected throughout all gastric cancer (GC) tissue samples, and its level of expression was higher in GC samples compared to nearby non-cancerous tissues (Figure 9A). Figure 9B shows that GC patients’ plasma samples have higher levels of CEBPA-AS1 compared to healthy subjects, which suggests that CEBPA-AS1 is a potential biomarker for the detection of GC [329].

According to current research, exosomes are essential for encouraging the epithelial–mesenchymal transition (EMT) in cancer cells. When nasopharyngeal tumor cells infected by Epstein–Barr virus have large numbers of exosomes containing significant amounts of LMP1 and HIF1α, this induces EMT and increases Twist expression, resulting in an increasingly invasive behavior in the cells that receive the infection [330]. The transcription of LMP1, an EBV oncogene that stabilizes HIF1α by reducing lysosomal breakdown and triggering EMT pathways, is required for this rise [331,332]. Exosomal miRNAs, including miR-23a, bolster TGF-β’s influence on EMT promotion. Tumor-derived exosomes from EMT-affected tumor cells can induce EMT-like characteristics in nearby cells, emphasizing their importance in the development and spread of cancer [333,334]. Tumor-derived exosomes influence infiltration into blood arteries directly and facilitate migration via extravasive migration (EMT). TDEs release miR-105, that in cancerous breast models breaks down tight junctions, an innate defense towards metastasis. Exosomal protein miR-105 is a predictive marker for the emergence of metastases that is found in the serum of individuals with breast carcinoma [335]. According to the findings by Zhang et al., tumor cells that express PTEN normally lose PTEN when they spread to brain tissue, yet not to the remaining organs. This decreased levels is controlled by brain astrocyte microRNAs, which is reversible. PTEN depletion can be restored by reducing PTEN-targeting microRNAs or preventing the production of the exosomes in the astrocyte, which will inhibit metastasis to the brain. The depletion of adaptive PTEN causes a spike in the release of chemokine CCL2 that attracts myeloid cells and promotes the development of tumor cells. Thus, for brain cancer metastases to occur, exosomes produced from astrocytes are necessary, as they rely on exosomal miRNA inhibiting PTEN [336]. Exosomal miR-21 is a diagnostic tool for lung carcinoma and aggressive melanoma, and it corresponds to an associated cancer spread and resurgence in cancer of the esophageal [337,338,339]. These results demonstrate the various functions of certain exosomal miRNAs in the development of malignancy. The tumor milieu is profoundly impacted by tumor-derived exosomes, which induce mesenchymal remodeling and turn mesenchymal stem cells into cancer-associated fibroblasts. The cancer-associated fibroblasts communicate back and forth with cancerous cells, as evidenced by their distinct hyaluronic acid layer and elevated expression of α-SMA [340]. TDEs facilitate such transformations by inducing modifications in the pericellular environment. Through the release of TGF-β1, CAFs trigger the EMT by triggering the TGF-β1-SMAD signaling cascade [341]. According to the findings by Webber, TGFβ1, which is an element of exosomes that are released by cancer cells, is essential for the advancement of malignancy. Exosomes promote angiogenesis and accelerate tumor development by inducing TGFβ1-dependent fibroblast transformation. Myofibroblasts produced by soluble TGFβ1 do not exhibit characteristics that promote tumor growth or angiogenesis. Rab27a’s removal of exosomes stops tumor development and differentiation, suggesting that exosomal TGFβ1 is necessary for the creation of stroma that promotes the growth of tumors. Myofibroblast markers such as αSMA and EDA-Fibronectin were found to be considerably overexpressed in lung fibroblasts when exposed to exosomes that are TGFβ positive Du145 cells. Growth factor release was changed; sTGFβ1 more effectively increased PDGF-AA and IGFBP-3, whereas exosomes selectively increased uPA and HGF. Unlike soluble TGFβ, exosomes that cause TGFβ1-dependent differentiating into unique myofibroblasts. As per the study, cancer exosomes containing TGFβ produce stroma that promotes tumor growth and angiogenesis via fibroblast differentiation. For cellular responsiveness and pathological alterations, TGFβ1’s biophysical shape is essential. Stomatogenesis is regulated by exosomal TGFβ, which makes up just 20% of the secretory system of cancer cells. The stromal growth-promoting effect is reduced by a defective exosome release [341]. For stromal differentiation to be driven toward a phenotype linked with cancer, exosome TGF-β1 is essential. Additionally, CAFs generate exosomes that on their own, boost migration potential and activate the pathway mediated by Wnt [342].

### 4.10. Lipids as Cancer Biomarkers

**Figure 10:**
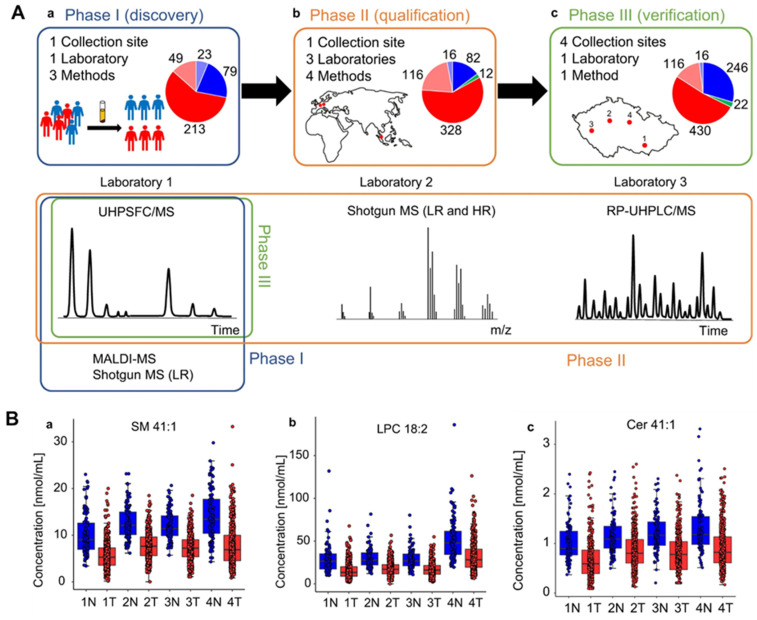
(**A**) Distinguishing PDAC patients (T = Tumor, red) from healthy controls (N = Normal, blue) and pancreatitis patients (Pan, green) via lipidomic profiling of human serum using various mass spectrometry methods. (**a**) Phase I involved analyzing 364 samples (262T + 102N) with ultrahigh-performance supercritical fluid chromatography/ mass spectrometry (UHPSFC/MS), shotgun MS (LR), and matrix-assisted laser desorption/ionization (MALDI-MS). These samples were divided into training (213T + 79N) and validation (49T + 23N) sets. (**b**) Phase II extended to 554 samples (444T + 98N + 12 Pan), divided into training (328T + 82N + 12 Pan) and validation (116T + 16N) sets. (**c**) In Phase III, 830 samples (546T + 262N + 22 Pan) were examined using UHPSFC/MS. These samples were split into training (430T + 246N + 22 Pan) and validation (116T + 16N) sets. LR = low resolution; HR = high resolution; RP = reversed-phase. (**B**) Representative box plots showing the concentration of lipids normalized using NIST documentation measured in patients with pancreatic ductal adenocarcinoma (PDAC) (443T) and control participants (95N) among both males and females: (**a**) sphingomyelins (SM) 41:1, (**b**) lysophosphatidylethanolamine (LPC) 18:2, and (**c**) ceramides (Cer) 41:1 [349].

Lipids serve a wide range of crucial functions in cells. Chemical energy storage, signaling, and structural stability for biomembranes are the most apparent functions. All these mechanisms are vital for cancer growth and metastasis to occur. Cancer arose as a reaction to damage due to changes in metabolism, including alterations in mitochondrial respiration and abnormal lipid production [343,344]. Multiple investigations have demonstrated that tumor cells exhibit dysregulated lipid metabolism, indicating that abnormalities in blood lipidome potentially promote tumor progression. Cardiolipins are exclusively found in the inner mitochondrial membrane among the lipids discovered in cells [345,346]. In comparison to healthy people, patients with lung cancer were reported as having higher levels of lipids such as sphingomyelin and lysophosphatidylethanolamine [347,348]. Figure 10 reveals that the alteration of ceramides (Cer), sphingomyelins (SM), and (lyso)phosphatidylcholines (LPC) can be utilized as potential prognostic markers for pancreatic ductal adenocarcinoma (PDAC) [349]. Jiang and colleagues reported the lipid species that could be utilized as markers for early detection of breast cancer. In comparison to healthy controls, researchers noticed higher amounts of Phytosterol Diosgenin (DG), and Phosphatidylcholines (PC) in breast cancer samples. The level of Phosphatidylethanolamine (PE) was shown to be lower in breast cancer samples [35]. Prostate cancer patients have a 2.7-fold elevation in Lysophosphatidylcholine (LPC) relative to healthy subjects, according to Zhou and colleagues [36].

Cellular biomembranes, which are made up of protein and lipid molecules, play an important part in many cellular functions. Membrane lipids, which include sterols, phospholipids, and sphingolipids, have an impact on the fluidity of the membrane, microdomain development, and cellular processes. Cell growth, inflammatory processes, the immune system, and apoptosis are all regulated by them. Lipids, such as phosphatidylethanolamine (PE), are required for cellular division, cytokinesis, and cytoskeletal structure [350]. These mechanisms are increased in cancer cells, resulting in uncontrolled proliferation. Lipids give significant details on malignant development [351,352,353,354,355,356]. For instance, Phosphorylated phosphoinositides (PIPs) are glycerophospholipids that govern cell development, growth, and movement. Because of their significance in malignancies such as breast, prostate, colon, thyroid, and ovarian malignancies, they are very essential in the biology of tumors [357,358,359,360,361,362]. Abnormalities in the PI3K signaling pathway can result in constitutive stimulation, whereas mutations in the lipid phosphatase PTEN, which is responsible for dephosphorylating PIPs, can result in ineffective phosphatase activity and elevated amounts of these lipids. In malignant tissues, downstream signaling via Akt is dysregulated, and PIs impact guanine nucleotide exchange factors for Rho GTPases, controlling the transition to malignancy. PIs and their metabolites are primary markers of cell signaling, and abnormalities can result in impaired cellular functioning and disease [363,364,365,366,367]. There are other lipids that have roles in different cancers, as shown in Table 6 below.

## 5. Clinical Classification of Cancer Biomarkers

### 5.1. Screening and Diagnostic Biomarkers

Screening and diagnostic cancer biomarkers are essential for the early detection of cancer, allowing for timely interventions and improved patient outcomes. Here are some examples of screening and diagnostic cancer biomarkers used for early detection:

Prostate-Specific Antigen (PSA) for Prostate Cancer: PSA is a widely used biomarker for screening and diagnosing prostate cancer. Elevated levels of PSA in blood samples can indicate the presence of prostate cancer. PSA testing is commonly performed in combination with other diagnostic tools to assess the risk of prostate cancer and guide further investigations [377].

Fecal Occult Blood Test (FOBT) for Colorectal Cancer: FOBT is a non-invasive screening test that detects hidden blood in the stool, which may indicate the presence of colorectal cancer or precancerous polyps. FOBT can help identify individuals who may require further diagnostic evaluations, such as colonoscopy [378].

Carcinoembryonic Antigen (CEA) for Colorectal, Lung, and Other Cancers: CEA is a biomarker commonly used in the diagnosis and monitoring of colorectal cancer [379]. Elevated CEA levels in blood samples can also indicate the presence of other cancers, such as lung, pancreatic, and breast cancers. CEA testing is often used in combination with imaging studies and other diagnostic tools.

CA-125 for Ovarian Cancer: CA-125 is a biomarker primarily used for ovarian cancer screening and monitoring. Elevated levels of CA-125 in blood samples can suggest the presence of ovarian cancer, although it is not specific to this disease and can be elevated in other conditions [380]. CA-125 testing is often combined with imaging studies and clinical evaluation.

Human Papillomavirus (HPV) Testing for Cervical Cancer: HPV testing is used for the screening and early detection of cervical cancer. Certain high-risk HPV strains are strongly associated with the development of cervical cancer. HPV testing, along with Pap smears or liquid-based cytology, helps identify women at risk and guide subsequent management and follow-up [381].

**Figure 11:**
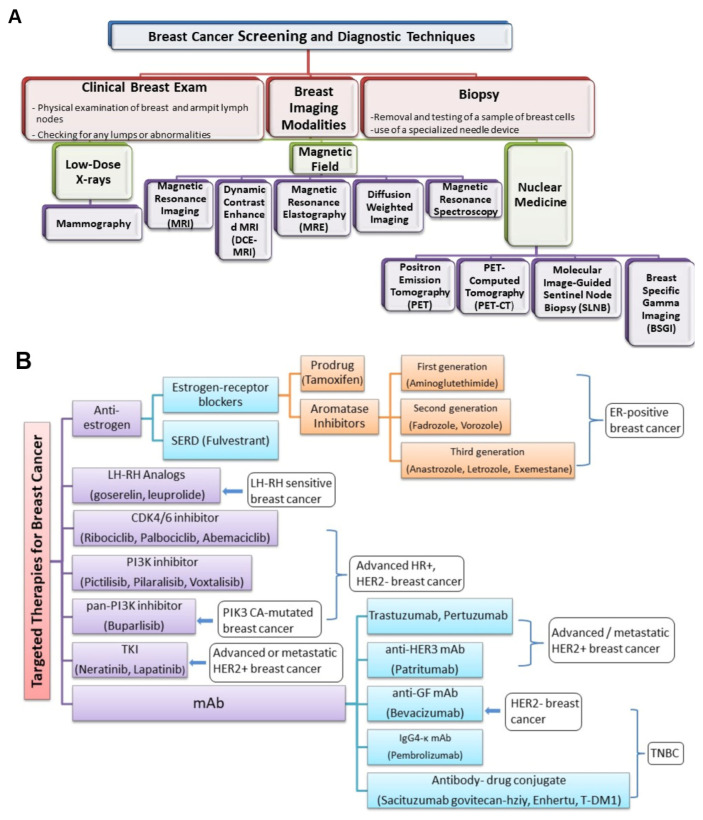
(**A**) A visual representation of the numerous imaging methods that can be used to diagnose breast cancer. (**B**) New targeted medicines approved by the FDA for the treatment of molecular subtypes of breast cancer [383].

Breast Imaging and Mammography for Breast Cancer: While not a specific biomarker, mammography and breast imaging techniques are crucial for the early detection of breast cancer (Figure 11A). Regular mammograms can help identify abnormalities, such as calcifications or masses, allowing for early diagnosis and prompt treatment [382].

### 5.2. Prognostic Biomarkers

#### 5.2.1. Predicting Disease Progression and Patient Outcomes

Prognostic biomarkers in cancer are used to predict the likely outcome or prognosis of a patient’s disease, including the likelihood of disease progression, survival rates, and response to treatment (Figure 11B). Here are some examples of prognostic biomarkers for different types of cancers:

Breast Cancer:
  a. Hormone Receptor Status: The presence or absence of estrogen receptor (ER), progesterone receptor (PR), and human epidermal growth factor receptor 2 (HER2) can help determine the prognosis and guide treatment decisions [384].
  b. Ki-67: A high expression of the Ki-67 protein, a marker of cellular proliferation, is associated with more aggressive breast cancer and poorer prognosis [385].
  c. Oncotype DX: A genomic test that analyzes the expression of a panel of genes to predict the risk of recurrence and guide the use of chemotherapy in early-stage breast cancer [386].
Colorectal Cancer:
  a. Microsatellite Instability (MSI): Tumors with high levels of MSI have a better prognosis and are associated with a higher response rate to immune checkpoint inhibitors [387].
  b. Carcinoembryonic Antigen (CEA): Elevated levels of CEA in the blood are associated with advanced disease and poorer prognosis in colorectal cancer [388].
  c. BRAF V600E Mutation: Patients with colorectal cancer harboring this mutation have a worse prognosis and may respond differently to certain treatments [389].
Lung Cancer:
  a. EGFR Mutation: Non-small-cell lung cancer (NSCLC) patients with EGFR mutations tend to have a better prognosis and may respond well to targeted therapies [390].
  b. ALK Rearrangement: NSCLC patients with ALK gene rearrangements have a better prognosis and are highly responsive to ALK inhibitors [391].
  c. PD-L1 Expression: Higher levels of PD-L1 expression in tumor cells are associated with a better response to immune checkpoint inhibitors in NSCLC [392].
Prostate Cancer:
  a. Prostate-Specific Antigen (PSA): PSA levels in the blood can provide information about the prognosis of prostate cancer, with higher levels often indicating a worse prognosis [393].
  b. Gleason Score: This scoring system evaluates the histological appearance of prostate cancer cells and helps predict the aggressiveness and prognosis of the disease [394].
  c. Androgen Receptor (AR) Expression: High levels of AR expression in prostate cancer cells are associated with a worse prognosis and resistance to androgen deprivation therapy [395].

#### 5.2.2. Tumor Staging and Grading Systems

Prognostic biomarkers play a crucial role in tumor staging and grading systems, providing valuable information about the aggressiveness of the tumor and the likelihood of disease progression. Here are some examples of prognostic biomarkers used in tumor staging and grading systems for different types of cancers:

Breast Cancer:
  a. Estrogen Receptor (ER) and Progesterone Receptor (PR) Status: ER-positive and PR-positive breast cancers tend to have a better prognosis compared to ER-negative and PR-negative tumors [396].
  b. Human Epidermal Growth Factor Receptor 2 (HER2) Expression: HER2-positive breast cancers are associated with a more aggressive disease and poorer prognosis [397].
  c. Ki-67: High levels of Ki-67, a marker of cellular proliferation, indicate a more aggressive tumor and are associated with poorer prognosis [385].
Prostate Cancer:
  a. Gleason Score: The Gleason scoring system evaluates the microscopic appearance of prostate cancer cells, with higher scores indicating a more aggressive tumor and poorer prognosis [398].
  b. Prostate-Specific Antigen (PSA) Velocity: The rate of change in PSA levels over time can help predict the risk of disease progression and metastasis [399].
  c. PTEN Loss: The loss of the PTEN gene, which regulates cell growth and division, is associated with a higher Gleason score and more aggressive prostate cancer [400].
Colorectal Cancer:
  a. Microsatellite Instability (MSI): Tumors with high levels of MSI are associated with a better prognosis and a lower risk of disease recurrence.
  b. BRAF V600E Mutation: Colorectal cancer patients with the BRAF V600E mutation have a worse prognosis and a higher likelihood of disease recurrence.
  c. KRAS Mutation: Specific KRAS mutations can indicate a more aggressive tumor and a poorer response to certain treatments [401].
Lung Cancer:
  a. TNM Staging: The TNM system incorporates tumor size (T), lymph node involvement (N), and metastasis (M) to determine the stage of lung cancer and predict prognosis.
  b. Epidermal Growth Factor Receptor (EGFR) Mutation: EGFR mutations are associated with a better prognosis and higher response rates to targeted therapies in lung cancer.
  c. ALK Rearrangement: Lung cancer patients with ALK gene rearrangements have a better prognosis and are highly responsive to ALK inhibitors.

### 5.3. Predictive Biomarkers

Predictive biomarkers in cancer are used to identify patients who are likely to respond positively or negatively to a specific treatment. These biomarkers help guide treatment decisions and optimize therapeutic strategies. Here are some examples of predictive biomarkers for cancer:

HER2 Status in Breast Cancer: HER2 overexpression or amplification in breast cancer is a predictive biomarker for response to HER2-targeted therapies such as trastuzumab and pertuzumab [402]. Patients with HER2-positive breast cancer tend to have a better response and improved outcomes with these targeted treatments.

EGFR Mutations in Non-Small Cell Lung Cancer (NSCLC): Specific mutations in the epidermal growth factor receptor (EGFR) gene in NSCLC, such as EGFR exon 19 deletions or the L858R mutation, are predictive biomarkers for response to EGFR tyrosine kinase inhibitors (TKIs) like gefitinib, erlotinib, and osimertinib [403]. Patients with these mutations are more likely to benefit from EGFR TKI therapy.

ALK Rearrangements in Lung Cancer: Rearrangements involving the anaplastic lymphoma kinase (ALK) gene in NSCLC are predictive biomarkers for response to ALK inhibitors like crizotinib, alectinib, and brigatinib [404]. Patients with ALK-positive lung cancer tend to have a higher response rate and longer progression-free survival with ALK-targeted therapies.

MSI-H/dMMR Status in Colorectal Cancer: Microsatellite instability-high (MSI-H) or deficient mismatch repair (dMMR) status in colorectal cancer is a predictive biomarker for response to immune checkpoint inhibitors like pembrolizumab and nivolumab [405]. Patients with MSI-H/dMMR tumors have shown significant responses and durable benefits with immune checkpoint blockade [406].

BRAF V600E Mutation in Melanoma: The BRAF V600E mutation in melanoma is a predictive biomarker for response to BRAF inhibitors (e.g., vemurafenib and dabrafenib) and MEK inhibitors (e.g., trametinib and cobimetinib) [407]. Patients with BRAF-mutant melanoma have shown improved response rates and progression-free survival when treated with targeted therapies.

ERCC1 Expression in Lung Cancer: Excision repair cross-complementation group 1 (ERCC1) expression levels in NSCLC have been explored as a predictive biomarker for response to platinum-based chemotherapy [408]. A low ERCC1 expression has been associated with improved response and survival outcomes in patients receiving platinum-based regimens.

## 6. Conventional Cancer Diagnostic Modes

Diagnosing cancer at its earliest stages provides the best chance for a better prognosis. Studies have shown that screening tests can save lives by diagnosing cancer early. There are several approaches to detecting cancer such as imaging tests, biopsy, lab tests, and physical examination.

The lab tests entail looking for biomarkers in blood or tissue specimens. The presence of elevated or inadequate amounts of specific substances in the body might indicate the existence of a malignancy. During the physical examination, the physician may look for aberrations in the body such as the presence of lumps, organ enlargement, and an alteration in skin tone as the signs of cancer. A biopsy is the type of cancer detection technique where sample of cells are being collected for laboratory analysis. A sample can be collected in a multitude of methods including endoscopy and needle insertion. Non-invasive imaging examinations are another mode of investigation which enables the physician to inspect the internal organs. A magnetic resonance imaging (MRI), computed tomography (CT) scan, positron emission tomography (PET) scan, bone scan, ultrasound, and X-ray are some of the imaging methods are being conducted to diagnose cancer. The various types of imaging testing utilize imaging biomarkers which play an important role in the clinical staging of cancer (TNM staging) and the routine management of individuals suffering from cancer. The clinically relevant imaging biomarkers and their role have been listed in Table 7.

## 7. Emerging Technologies and Techniques

### 7.1. Liquid Biopsy

**Figure 12:**
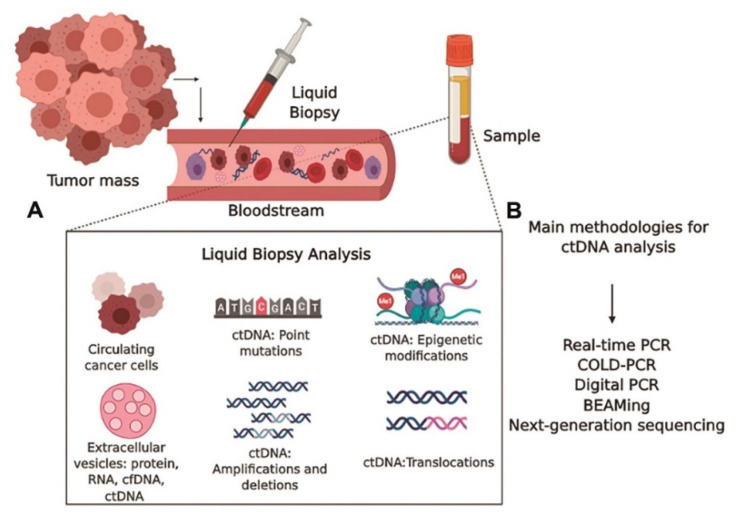
Liquid biopsy analysis [413]. (**A**) Liquid biopsy analysis involves the examination of circulating cancer cells, circulating tumor DNA (ctDNA), and extracellular vesicles containing proteins, RNA, ctDNA, and cell-free DNA (cfDNA) from both primary and secondary tumor sites. This approach is considered a potential cancer biomarker, enabling the quantification of ctDNA levels and the detection of (epi)genetic alterations. (**B**) Methods employed for ctDNA analysis encompass real-time PCR, BEAMing (beads, emulsion, amplification, and magnetics), coamplification at lower denaturation temperature PCR (COLD-PCR), digital PCR, and next-generation sequencing.

Liquid biopsy is a minimally invasive diagnostic procedure that involves the examination of numerous elements found in physiological fluids like blood or urine, including exosomes, circulating tumor cells, cell-free DNA (cfDNA), and proteins. With the help of this method, early cancer detection, treatment monitoring, and the discovery of potential therapeutic targets are made possible through insights into a patient’s molecular profile. Figure 12 provides an illustration of the methods used in liquid biopsy analysis. In this method, a single blood sample’s cfDNA/ctDNA profile is made up of both wild-type and genetically and epigenetically changed DNA fragments released by various tissues and organs through various pathways [413]. The advantages and limitations of liquid biopsy, as well as the companies offering these technologies, are listed in Table 8.

### 7.2. Single-Cell Analysis

Single-cell analysis has revolutionized our understanding of tumor heterogeneity by enabling the characterization of individual cells within a tumor. Here is how single-cell analysis helps to characterize tumor heterogeneity:

Identifying Subpopulations: Single-cell analysis allows the identification of distinct subpopulations of cells within a tumor. By analyzing the transcriptomic or genomic profiles of individual cells, researchers can identify and classify different cell types or states within the tumor. This reveals the heterogeneity in gene expression patterns, signaling pathways, and functional characteristics among tumor cells. 

Uncovering Clonal Diversity: Tumors are composed of clonal cell populations with genetic alterations acquired during tumor evolution. Single-cell genomic sequencing techniques, such as single-nucleus sequencing or single-cell whole-genome sequencing, can identify and characterize somatic mutations, copy number variations, and chromosomal rearrangements in individual cells. This helps to reveal clonal diversity and understand the evolutionary trajectory of the tumor. 

Profiling Transcriptomic Variability: Single-cell RNA sequencing (scRNA-seq) enables the profiling of gene expression patterns in individual cells. This allows the identification of different transcriptional states, gene regulatory networks, and functional states within the tumor. By analyzing the transcriptomic variability, researchers gain insights into the cellular heterogeneity, cell lineage relationships, and potential cell subpopulations with distinct biological properties. 

Assessing Protein Expression and Signaling: Techniques such as immunohistochemistry (IHC) and mass cytometry (CyTOF) at the single-cell level enable the characterization of protein expression profiles and signaling pathways in individual cells. This helps to understand the heterogeneity in protein expression, cellular phenotypes, and the activation of key signaling molecules within the tumor microenvironment. 

Mapping Spatial Heterogeneity: Spatial transcriptomics and imaging-based single-cell analysis techniques allow the assessment of cellular heterogeneity in the context of the tumor microenvironment. By characterizing the spatial distribution of different cell types, gene expression patterns, or immune cell infiltrates, researchers can unravel the spatial organization and heterogeneity of tumor cells within the tissue architecture. By combining these single-cell analysis approaches, researchers can comprehensively characterize the complexity and heterogeneity of tumors at the cellular level. This deeper understanding of tumor heterogeneity has implications for predicting treatment response, identifying therapy-resistant cell populations, and developing personalized treatment strategies in cancer patients.

### 7.3. Artificial Intelligence and Machine Learning

Artificial intelligence (AI) and machine learning (ML) techniques have made significant contributions to various aspects of cancer research, diagnosis, treatment, and patient care. Here are some key ways AI and ML are used in cancer:

Image Analysis and Medical Imaging: AI and ML algorithms are used to analyze medical images, such as mammograms, CT scans, and histopathology slides. These algorithms can assist in early cancer detection, tumor segmentation, identifying suspicious lesions, and predicting treatment response. Deep learning models have demonstrated remarkable accuracy in image-based cancer diagnosis. 

Genomic Analysis: AI and ML techniques are applied to genomic data analysis, including DNA sequencing and gene expression profiling. These algorithms can identify genomic alterations, mutations, and biomarkers associated with specific cancer types, helping in diagnosis, prognosis, and personalized treatment selection. 

Clinical Decision Support: AI and ML models can aid clinicians in making more informed decisions regarding cancer treatment plans. These models leverage patient data, such as medical records, imaging results, and genetic profiles, to provide personalized treatment recommendations, predict treatment outcomes, and optimize treatment strategies. 

Drug Discovery and Development: AI and ML are utilized in the early stages of drug discovery to identify potential drug targets, predict drug interactions, and design novel compounds. These techniques can also assist in drug repurposing by analyzing large-scale datasets and identifying existing drugs that may be effective against specific cancer types. 

Precision Medicine: AI and ML algorithms enable precision medicine approaches by integrating patient-specific data, including clinical, genomic, and imaging information. These models help identify patient subgroups that are more likely to respond to specific treatments, thus guiding personalized treatment selection and improving patient outcomes. 

Data Integration and Knowledge Extraction: AI and ML techniques can integrate and analyze large-scale, heterogeneous datasets from various sources, including electronic health records, medical literature, and public databases. By extracting knowledge and patterns from these data, AI models can identify associations, predict disease outcomes, and generate new insights for cancer research. 

Prognosis and Risk Assessment: AI and ML models can predict cancer prognosis, recurrence risk, and patient survival outcomes based on clinical and molecular features. These predictions assist in treatment planning, patient counseling, and monitoring long-term outcomes.

FDA-approved AI

IDx-DR: IDx-DR is an AI-based software that received FDA approval in 2018 for the autonomous detection of diabetic retinopathy in retinal images. While not specific to cancer, this highlights the application of AI in medical imaging for disease detection and diagnosis.

Viz.AI Contact: Viz.AI Contact is an AI software that received FDA approval in 2018 for the identification and notification of potential large vessel occlusion strokes. While not specific to cancer, it demonstrates the use of AI in assisting with time-sensitive diagnoses and treatment decisions.

Paige.AI Pathology Software: Paige.AI is a digital pathology company that received FDA approval in 2019 for its AI-based software platform, which assists pathologists in analyzing and interpreting digital pathology images. Pathology plays a crucial role in cancer diagnosis, and AI tools like Paige.AI can aid in improving efficiency and accuracy in pathology workflows.

## 8. Challenges and Future Directions

Heterogeneity and Complexity: The heterogeneity and complexity of cancer require the comprehensive characterization and validation of biomarkers across different cancer types, stages, and molecular subtypes. The integration of multi-omics data, including genomics, transcriptomics, proteomics, and imaging, can provide a more holistic understanding of cancer and enable the identification of robust biomarker signatures that capture the intricacies of the disease.

Standardized Assay Platforms and Protocols: Variations in assay platforms, protocols, and data analysis methods hinder the reproducibility and comparability of biomarker measurements. The development of standardized protocols, reference materials, and quality control measures can ensure consistency across laboratories and studies. Collaborative efforts and data sharing initiatives can promote transparency and enable the establishment of best practices for biomarker measurement.

Clinical Relevance and Utility: Biomarker validation requires evidence of clinical relevance and utility to guide treatment decisions and improve patient outcomes. Conducting prospective clinical trials that incorporate biomarker-guided strategies can demonstrate the clinical utility and cost-effectiveness of biomarkers. Real-world evidence, such as electronic health records and patient registries, can provide valuable insights into the impact of biomarker-guided interventions on patient outcomes.

Validation in Diverse Patient Populations: Biomarker validation should encompass diverse patient populations to ensure generalizability and address healthcare disparities. Ensuring the representation of diverse populations in clinical trials and biomarker validation studies is crucial. The inclusion of underrepresented groups, such as different ethnicities, age ranges, and coexisting medical conditions, can improve the applicability and equity of biomarker-guided approaches.

Integrative Data Analysis and Machine Learning: Biomarker validation often requires the integration of complex, large-scale datasets and advanced data analysis techniques. Leveraging machine learning algorithms and integrative data analysis methods can enhance biomarker discovery and validation. Utilizing artificial intelligence approaches to identify patterns, correlations, and predictive models can accelerate the identification and validation of novel biomarkers.

Regulatory Approval and Clinical Adoption: The regulatory approval and clinical adoption of biomarkers require rigorous evidence of clinical validity, utility, and patient benefit. Collaborative efforts among researchers, clinicians, regulatory bodies, and industry stakeholders are essential. Streamlining regulatory processes, incorporating real-world evidence, and establishing clear pathways for biomarker integration into clinical practice can facilitate the efficient translation and adoption of validated biomarkers.

## Figures and Tables

**Table 1:** Comparison between different genetic biomarkers.

Genetic Biomarker	Cancer Type	Function/Use	Key Genes/Elements	Ref.
Mutations and Gene Alterations	Melanoma	Targeted therapy selection	BRAF V600E mutation	[117]
	NSCLC (Lung)	Sensitivity to EGFR inhibitors	EGFR mutations (e.g., exon 19 deletions, L858R)	[118]
	Colorectal	Affecting treatment response	KRAS mutations (30–40% cases)	[119]
	Breast/Ovarian	Guiding therapy selection	BRCA1/BRCA2 mutations	[70]
	Breast/Gastric	Indicating aggressive behavior	HER2 amplification/overexpression	[71]
	Gliomas	Diagnostic and prognostic markers	IDH mutations	[120]
Gene Expression Profiles	Breast	Recurrence prediction and treatment guidance	Oncotype DX (16 genes)	[121]
	Breast	Distant metastasis risk and treatment guidance	MammaPrint (18 genes)	[122]
	Breast	Subtype classification and hormone therapy	Prosigna (PAM50—50 genes)	[75]
	Prostate	Recurrence prediction after surgery	Decipher (Genomic Risk Score)	[123]
DNA	Various	Identification of oncogene alterations	p53, KRAS, APC, RAS, BRCA1/2, etc.	[124]
	Various	Detection of mismatch-repair gene mutations	Mismatch-repair gene mutations	[125]
	Various	Monitoring of circulating DNA	Tumor DNA in circulation	[126]
RNA	Various	Identification of miRNA markers	Various microRNAs in different cancers	[127]
	Lung	Detection of circular RNA (circRNA) markers	Hsa circ 0013958 in lung adenocarcinoma	[128]
Epigenetics	Various	Detection of DNA methylation in promoter regions	RASSF1A, p16, BRCA1, NKX2-6, SPAG6, PER1, ITIH5, etc.	[129]
	Various	Role of histone acetylation	Histone acetylation levels	[130]

**Table 2:** Protein biomarkers for different cancer diagnoses.

Protein Tumor Marker	Typical Concentration in Healthy Populations	Typical Concentration in Cancer Patients	Reference
Alpha-Fetoprotein (AFP)	<10 ng/mL	Elevated levels (>200–400 ng/mL) in hepatocellular carcinoma (HCC) and other cancers	[136]
Carcinoembryonic Antigen (CEA)	<3 ng/mL	Elevated levels in various cancers, including colorectal, lung, and pancreatic cancer	[137]
Prostate-Specific Antigen (PSA)	<4 ng/mL	Elevated levels in prostate cancer	[138]
CA-125	<35 U/mL	Elevated levels in ovarian and other gynecological cancers	[139]
CA 19-9	<37 U/mL	Elevated levels in pancreatic and other gastrointestinal cancers	[140]
CA 15-3	<30 U/mL	Elevated levels in breast cancer	[141]
CA 27.29	<40 U/mL	Elevated levels in breast cancer	[142]
Human Chorionic Gonadotropin (hCG)	<5 IU/L	Elevated levels in germ cell tumors, including testicular and ovarian cancer	[143]
Human Epidermal Growth Factor Receptor 2 (HER2)	Negative (score 0 or 1+ by immunohistochemistry)	Overexpression or amplification in HER2-positive breast and gastric cancer	[144]

**Table 3:** Cancer types and FDA-approved immunotherapies.

Cancer Type	Group of Patients Who May Benefit	FDA-Approved Immunotherapies	Reference
Melanoma	Advanced/metastatic melanoma	Pembrolizumab (Keytruda), Nivolumab (Opdivo), Ipilimumab (Yervoy)	[145,146,147,148]
Lung Cancer	Non-small-cell lung cancer (NSCLC)	Pembrolizumab, Nivolumab, Atezolizumab (Tecentriq), Durvalumab (Imfinzi), Combination therapies: Pembrolizumab + Chemotherapy	[149]
Head and Neck Cancer	Recurrent or metastatic squamous cell carcinoma	Pembrolizumab, Nivolumab	[150]
Bladder Cancer	Locally advanced or metastatic urothelial carcinoma	Atezolizumab, Pembrolizumab, Nivolumab	[151]
Kidney Cancer	Advanced or metastatic renal cell carcinoma	Nivolumab, Pembrolizumab, Axitinib + Pembrolizumab, Combination therapies: Avelumab + Axitinib	[152]
Hodgkin Lymphoma	Classical Hodgkin lymphoma	Pembrolizumab, Nivolumab	[153]
Colorectal Cancer	Microsatellite instability-high (MSI-H)/dMMR	Pembrolizumab, Combination therapy: Nivolumab + Ipilimumab	[154]

**Table 4:** Roles of various types of lamins in different cancer types.

Type of Cancer	Type of Lamin Involved	Gene Name	Phenotype of Lamin	Phenotype of Cancer	Ref.
Colorectal	Lamin A/C	LMNA/LMNC	Decreased expression of lamin A/C	High motility and recurrence	[213]
Pancreatic Cancer	Lamin B1	LMNB1	Overexpression of lamin B1	Invasiveness and poor prognosis	[206]
Gastrointestinal	Lamin A/C	LMNA/LMNC	Decreased lamin A/C expression	Invasiveness	[218]
Neuroblastoma	Lamin A/C	LMNA/LMNC	Decreased lamin A/C expression	Cell motility and invasiveness	[216]
Prostate	Lamin B	LMNB1	Increased expression of lamin B	Augmented aggressiveness and motility	[219]
Germ cell	Lamin C	LMNC	Increased expression of lamin C	Cell motility and invasiveness	[207]
Liver	Lamin B1	LMNB1	Increased expression of LMNB1	Cell motility and invasiveness	[220]
Lung	Lamin A/C	LMNA/LMNC	Increased lamin A/C expression	Increased migratory property	[221]
Breast	Lamin A/C	LMNA/LMNC	Decreased lamin A/C expression	Altered morphology and aneuploidy	[214,215]
Skin	Lamin A	LMNA	Increased lamin A expression	Increased migratory property	[222]

**Table 5:** Serum levels of galectins in malignant cancers compared to healthy conditions.

Type of Organ Systems	Distribution of Patient Studies on Galectins on Different Organ Systems	Types of Cancers	Subtypes	Galectin 1	Galectin 2	Galectin 3	Galectin 4	Galectin 7	Galectin 8	Galectin 9	Galectin 12	Ref.
Digestive	28.75%	Bile duct		-	-	Similar	-	-	-	-		[256,257]
		Colon		Increased	Similar	Increased	Decrease	Increased	Decreased			[256,258,259,260,261,262,263]
		Esophagus		-	-	-	-	Increased	-	-	-	[245,256]
		Gall bladder		-	-	Increased	-	-	-	-	-	[256,264]
		Gastric		-	Similar	Similar	-	Decreased	Similar	Decreased		[244,256,259,263,265,266]
		Liver		Increased	Decreased	Increased	Increased		Decreased	Decreased		[256,259,263,267,268,269,270,271]
		Pancreas		Increase	Similar	Increased	Increased	-	Decreased	Decreased		[256,259,263,272,273,274,275]
		Oral		Decreased	-	Decreased	-	-	-	-	-	[241,256]
		Tongue		Decreased		Decreased						[241,256]
Hematologic	13.1%	Lymphoid	Lymphoma	Increased	-	Increased	-	-	Increased	Increased		[256,276,277,278,279]
			B-cell lymphoma	-	-	Decreased						[256,280]
			Non-Hodgkin’s lymphoma	-	-	Similar	-	-	-	-		[229,256]
		Myeloid		Increased		Increased						[256,281]
Neural	6.3%	Brain				Increased						[256,282,283]
		Glioma		Increased	-	-	-	-	-	-		[256,284]
		Pituitary gland				Increased						[256,285]
Reproductive	22.4%	Breast		Increased	Decreased	Decreased		Increased	Increased			[246,256,259,263,286]
		Cervix		Increased		Decreased		Decreased		Decreased		[256,287,288,289,290]
		Ovarian		Increased	Increased	Decreased	Increased	-	-	-		[256,259,291,292,293]
		Prostate		Similar	-	Decreased	Decreased		Increased	Decreased	Decreased	[256,294,295,296,297]
		Uterus		Similar		Similar						[256,298]
Respiratory	13.9%	Larynx		Decreased		Decreased		Increased	Decreased			[241,248,256,263]
		Lungs		Increased	Similar	Increased	-	Similar	Increased	-	-	[231,233,256,259,299]
		Nasal cavity				Decreased	-	-	-	-	-	[256,300]
		Pharynx		Decreased	-	Decreased	-	-	Increased	Increased		[241,248,256,301]
Urinary	7.2%	Bladder		Increased	Similar	Increased	Increased	Similar	Similar	-		[229,232,256,259,263]
		Kidney		Increased	Similar	Decreased		Increased	Similar			[235,256,259,263,302]
Miscellaneous	8.4%	Skin	Basal cell carcinoma	Increased	Decreased	Increased	Decreased	Decreased	Decreased	Decreased		[239,256]
			Melanoma	-	-	Increased	-	-	-	-		[256,303]
			Squamous cell carcinoma	Decreased		Decreased						[239,241,256]
		Thyroid		Increased	Increased	Increased	-	Increased	-	-		[238,256,259]

**Table 6:** List of other lipids that may be involved in various types of cancer.

Lipids	Composition	Presence of the Side of Membrane	Cancer Types	Levels	Functions	Ref.
Phosphoinositide	Phosphate group, two fatty acid chain and inositol molecule	Primarily located inner leaflet	Breast, Prostate, Ovarian, Lung, and Gastric	Increased	Signaling for cell proliferation and motility	[363,364,365,366]
Cholesterol	Sterol composed of four hydrocarbon rings, and a hydroxyl group	Primarily located outer leaflet	Most types	Alteration	Controlling membrane structure	[368]
Prostaglandin	Five member ring with fatty acid, arachidonic acid	Primarily located inner leaflet	Most types	Secreted by cancer cells	Angiogenesis, anti-apoptosis, invasion	[369]
lysophosphatidic acid	Contains a glycerol backbone with a phosphoryl group	Primarily located outer leaflet	Ovarian	Increased	Enhanced metastasis	[370,371,372]
sphingosine 1 phosphate	Amphipatic lysophospholipid composed of a sphingoid extended chain and a phosphate molecule.	Primarily located outer leaflet	Most types	Secreted by cancer cells	Angiogenesis	[373,374,375,376]
Ceramides	Contains sphingosine and a fatty acid	Both leaflets	Breast, Prostate, and Ovarian	Decreased level	Cell cycle arrest and apoptosis	[368]

**Table 7:** A compilation of imaging markers and modalities that are currently being used for the early diagnosis of cancer.

Biomarker	Approach	Clinical Role	Clinical Phase	Reference
Breast morphology	Mammography	Breast cancer diagnosis	Translational gap 2	[37]
Clinical Tumor, Node, Metastasis (TNM) staging	MRI, CT, PET	Prognosis for all cancers	Translational gap 2	[409]
T-score (BMD − Reference BMD)/SD BMD = Bone mineral density SD = standard deviation	Dual-energy X-ray absorptiometry (DXA)	Safety marker; recommending bisphosphonates to individuals with breast cancer who are experiencing bone loss as a consequence of their treatment	Translational gap 2	[41]
Bone scan index (M/R) × C M = area of the metastasis R = area of the anatomical region where the metastasis is located C = coefficient reflecting the regional proportion of skeletal mass	Single-photon emission computed tomography (SPECT)	Prognosis for prostate cancer	Translational gap 2	[410]
Magnetic resonance imaging in breast screening (MARIBS) category	MRI	Determining the probability of breast cancer in individuals with genetic predisposition, including mutations in BRCA1 or BRCA2	Translational gap 2	[411]
^99m^Tc-etarfolatide FR+ FR+ = folate receptor-positive	SPECT	Diagnosis for platinum-resistant ovarian cancer	Companion diagnostics evaluated by European Medicines Agency (EMA)	[43]
Mucosal abnormalities	White-light imaging	Diagnosis in melanoma	-	[412]

**Table 8:** Advantages, limitations, and companies offering liquid biopsy technologies.

Liquid Biopsy Technology	Advantages	Limitations	Company Offering
Circulating Tumor DNA (ctDNA) Analysis	- Non-invasive, requires a simple blood draw - Can detect tumor-specific genetic alterations and monitor treatment response - Provides real-time tumor profiling	- Detection sensitivity can be affected by tumor size and shedding of ctDNA - False positives and negatives are possible - Requires specialized equipment and expertise	- Guardant Health - Personal Genome Diagnostics (PGDx) - Foundation Medicine
Circulating Tumor Cells (CTCs) Analysis	- Non-invasive, requires a blood sample - Can capture intact tumor cells for molecular and genetic analysis - Potential for assessing cancer metastasis and drug resistance	- CTCs are rare in the bloodstream, making their isolation and analysis challenging - CTC heterogeneity and variability can affect accuracy - May require enrichment methods for better detection	- Menarini Silicon Biosystems - Epic Sciences - RareCyte
Extracellular Vesicle (EV) Analysis	- EVs carry diverse biomarkers, including proteins, nucleic acids, and lipids - Can be isolated from various bodily fluids, including blood and urine - EVs offer potential for early cancer detection and monitoring	- EV isolation and purification can be complex and require specialized protocols - Heterogeneity of EV subpopulations can complicate analysis - Standardization of EV analysis methods is ongoing	- Exosome Diagnostics - Aethlon Medical - Codiak Biosciences
Cell-Free DNA (cfDNA) Analysis	- Non-invasive, requires a blood sample - Can detect genetic alterations, including mutations, rearrangements, and copy number variations - Can monitor treatment response and minimal residual disease	- cfDNA levels can be low, requiring sensitive detection methods - Non-tumor-derived cfDNA can interfere with analysis - Detection of tumor-derived cfDNA can be challenging in early-stage cancers	- Grail - Natera - Resolution Bioscience

## Data Availability

Not applicable.
